# The Enhancer of split transcription factor Her8a is a novel dimerisation partner for Her3 that controls anterior hindbrain neurogenesis in zebrafish

**DOI:** 10.1186/1471-213X-11-27

**Published:** 2011-05-17

**Authors:** Katharine J Webb, Marion Coolen, Christian J Gloeckner, Christian Stigloher, Brigitte Bahn, Stefanie Topp, Marius Ueffing, Laure Bally-Cuif

**Affiliations:** 1 Zebrafish Neurogenetics Department, Helmholtz Zentrum München, German Research Center for Environmental Health, Ingolstädter Landstr. 1, D-85764 Neuherberg, Germany; 2 Department of Protein Science, Helmholtz Zentrum München, German Research Center for Environmental Health, Ingolstaedter Landstr. 1; D-85764 Neuherberg, Germany; 3 Division of Experimental Ophthalmology and Medical Proteome Center, Center of Ophthalmology, University of Tübingen, D-72076 Tübingen, Germany; 4 Zebrafish Neurogenetics Group, Laboratory of Neurobiology and Development (N&D), CNRS UPR 3294, Institute of Neurobiology Alfred Fessard, Avenue de la Terrasse, Building 5, F-91198-Gif-sur-Yvette, France; 5 Max Planck Institute for Psychiatry, Kraepelinstr. 2-10, 80804, Munich, Germany; 6 Institute for Developmental Genetics, Helmholtz Zentrum München, German Research Center for Environmental Health, Ingolstädter Landstr.1, D-85764 Neuherberg, Germany; 7 Genetics and Neurobiology of C. elegans, INSERM U1024, Institute of Biology of the Ecole Normale Supérieure, 46 rue d'Ulm, F-75005 Paris, France; 8 Roche Diagnostics GmbH, Nonnenwald 2, D-82377 Penzberg, Germany; 9 Eppendorf Instrumente GmbH, Barkhausenweg 1, 22339, Hamburg, Germany; 10 Zebrafish Neurogenetics Group, Laboratory of Neurobiology and Development (N&D), CNRS UPR 3294, Institute of Neurobiology Alfred Fessard, Avenue de la Terrasse, Building 5, F-91198 Gif-sur-Yvette, France

**Keywords:** zebrafish, primary neurogenesis, midbrain-hindbrain, Hairy/E(spl), Her/Hes

## Abstract

**Background:**

Neurogenesis control and the prevention of premature differentiation in the vertebrate embryo are crucial processes, allowing the formation of late-born cell types and ensuring the correct shape and cytoarchitecture of the brain. Members of the Hairy/Enhancer of Split (Hairy/E(spl)) family of bHLH-Orange transcription factors, such as zebrafish Her3, 5, 9 and 11, are implicated in the local inhibition of neurogenesis to maintain progenitor pools within the early neural plate. To better understand how these factors exert their inhibitory function, we aimed to isolate some of their functional interactors.

**Results:**

We used a yeast two-hybrid screen with Her5 as bait and recovered a novel zebrafish Hairy/E(spl) factor - Her8a. Using phylogenetic and synteny analyses, we demonstrate that *her8a *evolved from an ancient duplicate of *Hes6 *that was recently lost in the mammalian lineage. We show that *her8a *is expressed across the mid- and anterior hindbrain from the start of segmentation. Through knockdown and misexpression experiments, we demonstrate that Her8a is a negative regulator of neurogenesis and plays an essential role in generating progenitor pools within rhombomeres 2 and 4 - a role resembling that of Her3. Her8a co-purifies with Her3, suggesting that Her8a-Her3 heterodimers may be relevant in this domain of the neural plate, where both proteins are co-expressed. Finally, we demonstrate that *her8a *expression is independent of Notch signaling at the early neural plate stage but that SoxB factors play a role in its expression, linking patterning information to neurogenesis control. Overall, the regulation and function of Her8a differ strikingly from those of its closest relative in other vertebrates - the Hes6-like proteins.

**Conclusions:**

Our results characterize the phylogeny, expression and functional interactions involving a new Her factor, Her8a, and highlight the complex interplay of E(spl) proteins that generates the neurogenesis pattern of the zebrafish early neural plate.

## Background

Neurogenesis in the early vertebrate neural plate begins at stereotyped loci - termed proneural clusters -, which prefigure the localization of the earliest neuronal groups and the architecture of the primary embryonic neuronal scaffold. These proneural clusters consist of spatially defined progenitor groups engaged in active neurogenesis, within which committed precursors expressing higher levels of proneural genes (such as *neurogenin *or *achaete-scute*-like genes, respectively *neurog1 *and *ascl1 *in zebrafish) are singled out to differentiate first. An identical scaffold is found in all vertebrate embryos, highlighting the robustness and functional relevance of this organization [1-4]. Dissecting the regulatory cascades involved in this process is therefore of universal importance.

The control of neurogenesis progression within proneural clusters relies on Hairy/Enhancer of split (E(spl)) factors (Hes in mouse; Her in zebrafish). These transcription factors belong to the basic-helix-loop-helix (bHLH) family, characterized by a DNA-binding basic domain and an HLH domain composed of two alpha helices intervened by a loop of a few amino acids [5]. In addition, Hairy/Enhancer of split (E(spl)) factors contain an Orange domain, which is most probably involved in protein-protein interactions, and a WRPW C-terminal tetrapeptide, which mediates transcriptional repression (reviewed in [6,7]). During the so-called process of lateral inhibition, the expression of Notch ligands in committed precursors activates Notch signaling in neighbouring progenitors, which in turn induces expression of Hes/Her factors. The latter down-regulate proneural genes, hence maintaining Notch-receiving cells in a progenitor state. Reflecting the intermingled distribution of committed and transiently inhibited progenitors, the proneural and *E(spl) *genes are expressed in a salt-and-pepper fashion within proneural clusters. E(Spl) factors expressed in proneural clusters in zebrafish include *her4.1 *[8-10], *hes5/her15, her2 *and *her12 *[11]. In agreement with the lateral inhibition model, *her4.1 *expression is positively regulated by Notch, and inhibits expression of *neurog1 *[10].

Recent work has demonstrated that proneural clusters are delimited negatively, through a process of active neurogenesis suppression taking place in surrounding areas (reviewed in [12]). These "inhibited" areas, so-called "progenitor pools", are transiently maintained in an refractory state to be recruited in later events of neuronal production, and are organized as tight groups of adjacent cells at stereotyped positions within the neural plate. Major progenitor pools can be found at the presumptive midbrain-hindbrain boundary (MHB) [3,13] and in longitudinal stripes separating the columns of presumptive moto- and lateral neurons in the hindbrain, or moto-, inter- and sensory neurons in the spinal cord [11,14,15]. At the least, the MHB pool is maintained until adulthood in zebrafish, where it participates in the generation of adult-born neurons and oligodendrocytes [16]. Embryonic progenitor pools are characterised by the expression of a specific set of transcription factors, including Zic, BF1/Anf and Rx family members [17] as well as Hes/Her proteins. In zebrafish, the combinatorial expression of a distinct set of *her *genes - which to date includes *her3, her5, her9 *and *her11 *- characterises all progenitor pools [12], while in mouse the genes *Hes1, Hes3 *and *Hes5 *share sustained expression in adjacent cells of the MHB pool, for example [18-20]. These *her/Hes *genes exhibit functional similarities and have been implicated in progenitor pool maintenance: their misexpression inhibits neurogenesis, whereas loss-of-function causes premature expression of proneural genes in at least part of their expression domains [11,13,15,19-22]. In addition, *her3/5/9/11 *as well as *Hes1 *at the mouse MHB all demonstrate an irregular association with Notch: while Hes/*her *genes in proneural clusters are activated by Notch signaling, the expression of *her3/5/9/11 *and Hes1 at the MHB is controlled in a Notch-independent manner. The mechanisms accounting for these specific features remain unknown.

The HLH region of Hes/Her factors functions as a dimerisation domain, and the formation of hetero- and homodimers as well as further possible interactions through the Orange domain are key components of the specificity of the actions of these proteins. Heterodimerisation can involve closely related members of the Her/Hes family, or several different transcription factors or transcriptional cofactors [7]. In order to better understand the mechanism of action of Her factors expressed in progenitor pools, and the pathways regulating their activity, we performed a yeast two-hybrid screen using the HLH and Orange domains of Her5 as bait. This led to the recovery of Her8a, a novel Her factor of the Hes6 subfamily expressed in a broad manner at the presumptive midbrain-hindbrain domain of the early zebrafish neural plate. Morpholino-mediated knockdown and misexpression studies establish Her8a as a negative-regulator of neurogenesis playing an essential role in maintaining progenitor pools of rhombomeres (r) 2 and 4. *her8a *knockdown produces a similar phenotype to that of *her3 *knockdown, and co-purification demonstrates that Her8a dimerises with Her3. At the MHB however, we show that the predominant activity is exerted by the combination of Her3, 5, 9 and 11. Together, our results identify a new player in progenitor pool formation and highlight the region-specific combinatorial activity of E(spl) factors in this process.

## Results

### Identification of Her8a as a potential binding partner for Her proteins

To recover binding partners for Her proteins, we used a yeast two-hybrid screen where a 181-amino acid fragment of Her5 (excluding the basic domain and the WRPW motif) was screened against an 18-20 hpf embryo zebrafish library. This screen returned 280 positive clones, from a total of 76.1 million tested interactions. These 280 positive clones represented 75 unique protein-protein interactions. The quality of these interactions was graded using a PBS scoring system - where A is the highest score of confidence, B is very good, C is good, D is low and N/A is used when no score could be assigned (see Materials and Methods). Our screen returned 6 As, 9 Bs, 2 Cs, 49 Ds and 9 N/As (see Additional file 1, Table S1 for a detailed description of all recovered candidates). Gene ontology enrichment analysis of the recovered binding partners revealed an enrichment of proteins involved in protein transport and also heterodimerisation (see Additional file 2, Table S2). Among these candidates, we note the presence of 7 distinct Her factors, in agreement with the postulated capacity for protein heterodimerisation within this family [7]. As further indication of the validity of the assay, Her5 was found to bind with Her11 with a score of B, an interaction that had been shown previously in our laboratory [22]. Her8a, scored A and corresponding to a new E(spl) family member, proved very strongly expressed in the midbrain-hindbrain (MH) area (see below), and we consequently focused on this factor.

### Her8a is a new Hes6-like protein, the ortholog of which was lost in the mammalian lineage

**Figure 1:**
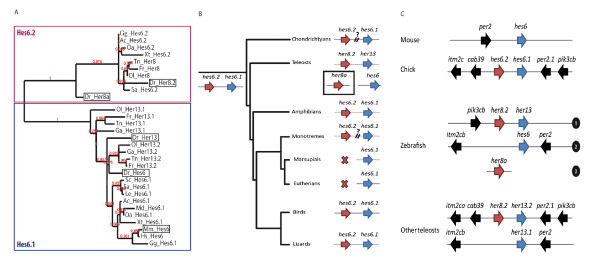
***her8a *encodes a Hes6-like E(spl) protein closely related to, but not directly orthologous to, mammalian Hes6**. **A. **Phylogenetic tree depicting protein relationship within the Hes6 subfamily, based on the bHLH and Orange domain sequences. Note the absence of Hes6.2 proteins in marsupials and eutherian mammals. In zebrafish, the closest relative to Her8a is Her8.2, and the closest relative to Her13 is Hes6. **B. **Genomic organization of *Hes6*-like genes through evolution, confirming that the generation of the two *Hes6.1 *and *Hes6.2 *genes is ancestral and that *Hes6.2 *was secondarily lost within the mammalian taxa subsequent to the divergence of marsupials and eutherian mammals from monotremes. "?" indicates cases where genomic linkage cannot be resolved at present, in the absence of a genome sequence for the corresponding species. **C. **Genomic organization of the areas surrounding *Hes6*-like genes in teleost species compared to mouse. With the exception of zebrafish, for all of the teleosts studied, synteny analyses suggest a secondary loss of the *hes6.2 *duplicate (missing from locus 2) after the duplication of locus 1. In zebrafish, this gene (*her8a*) has been relocated to a third locus on a distinct chromosome. 1-3: genomic loci.

The predicted Her8a protein displays a bHLH and an orange domain, and harbors a WRPW motif at its C-terminus - characteristics common to the Hairy/E(spl) family. Sequence comparisons of the bHLH domain of this family classify Her8a, together with zebrafish Her13 (previously Her13.1), Hes6 (previously Her13.2) and Her8.2, within the subfamily showing highest homology to mouse Hes6 [23]. Proteins of this subfamily exhibit a shortened loop when compared to other Hairy-E(spl) members [24], such as mouse Hes1 and Hes5 (Additional file 3, Figure S1). Hes6-like proteins also share substantial similarity outside the bHLH domains (Additional file 3, Figure S1), allowing their phylogeny to be studied using extended protein sequences. This confirmed the existence of two subfamilies of Hes6-like proteins, Hes6.1 and Hes6.2, comprising respectively Her13/Hes6 and Her8a/Her8.2 (Figure 1A) (and see [23]), encoded by gene pairs (Figure 1B). Importantly, it also resolved for the first time their relationship with the single mammalian Hes6 protein, as sequence alignments directly assign mammalian Hes6 to the Hes6.1 subfamily (Figure 1A). *hes6.2*-like genes are found neither in eutherian mammals nor in marsupials, but exist in all other phyla, suggesting a late secondary loss of this gene shortly after the divergence of eutherians and marsupials from the monotreme lineage. Finally, and as expected from the whole genome duplication undergone in the teleost fish lineage subsequent to its divergence from other vertebrates [25], followed by secondary gene loss, teleost species exhibit three or four *Hes6*-like genes. Synteny analyses (Figure 1C) indeed identify a conserved orthologous gene pair (locus 1) as well as a conserved duplicate (locus 2). This duplicate only contains the *hes6.1*-like member and, based on the situation in other vertebrates, most likely lost the *hes6.2*-like gene. Strikingly, in zebrafish, the latter gene (*her8a*) was kept and transferred onto a different genomic location (locus 3). Together, these results indicate that *her8a *is the ortholog of *her8.2 *and that it is located in a unique genomic setting compared to other related genes. It also assigns *her8a *to a subfamily of *Hes6*-like genes that is closely related to but not directly orthologous to mammalian *Hes6*.

### *her8a *is expressed across progenitor pools and proneural clusters in the anterior neural plate

**Figure 2:**
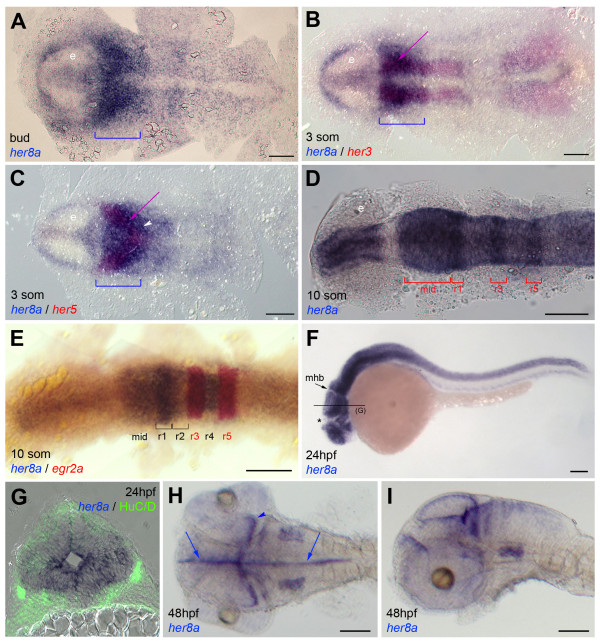
**Embryonic expression of *her8a***. Expression is revealed by whole-mount in situ hybridization at the stages indicated (som: somites, hpf: hours post-fertilization) together with other positional marker genes (B,C,E, color-coded) or proteins (G, immunocytochemistry for the neuronal marker HuC/D in green). A-E and H are dorsal views of flat-mounted embryos, F,I are lateral views, all embryos are viewed anterior left. G is a cross section of a 24hpf embryo at midbrain levels (as indicated in F). At early neural plate stages, the domain of strongest *her8a *expression covers the mid- and anterior hindbrain (bracket in A-C). It overlaps the presumptive midbrain-hindbrain boundary (*her5*-positive, purple arrow in C) and the progenitor pools separating medial and lateral hindbrain neurons (*her3*-positive, purple arrow in B). It extends into rhombomere 2 (white arrowhead in C), more posterior rhombomeres being more weakly labeled. At 10 somites, expression in the midbrain is maintained. It resolves in stripes in the rhombencephalon (D,E). It avoids the midbrain-hindbrain boundary and zona limitans intrathalamica (asterisk). From 24 hpf onwards, *her8a *characterizes the ventricular zone and progenitor domains of the neural tube (G-I, arrows in I point to the ventricular zone and the arrowhead points to the progenitor domain of the optic tectum). Abbreviations: e: eye field, mid: presumptive midbrain, mhb: midbrain-hindbrain boundary, r: rhombomere. Scale bars: 100 μm.

**Figure 3:**
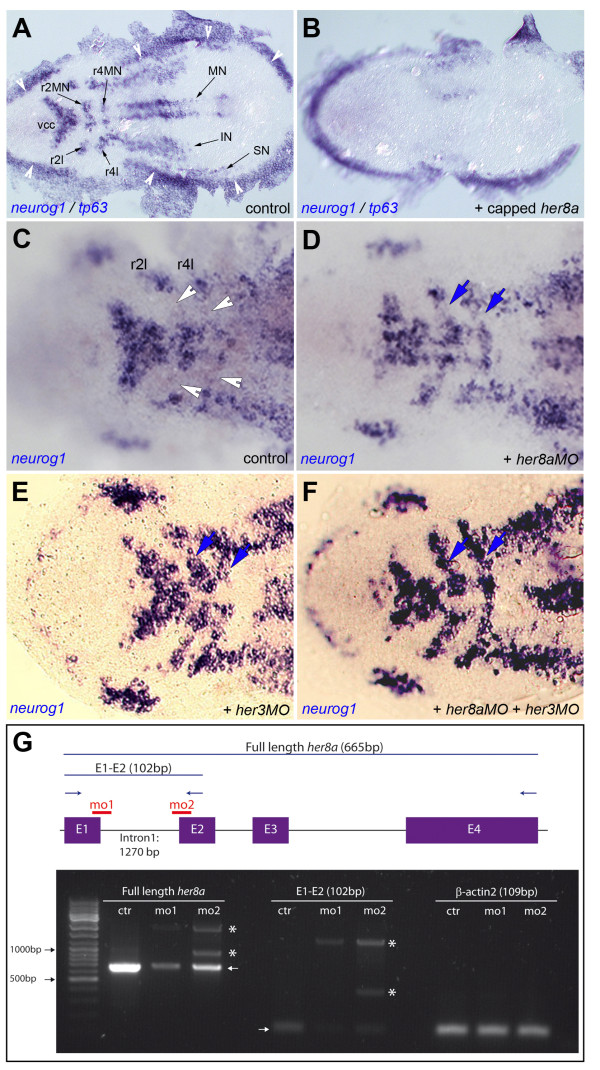
**Like Her3, Her8a activity maintains the non-neurogenic areas of rhombomeres 2 and 4**. **A,B. ***neurog1 *expression highlights the proneural clusters at the 3-somite stage (black arrows in A) and is eliminated upon *her8a *overexpression (B, embryo injected with *her8a *capped mRNA). Expression of *tp63*, which highlights the neural plate border (white arrowheads in A), is unchanged. **C-F. **Compared expression of *neurog1 *in control embryos (C) and embryos injected with *her8aMO *(D), *her3MO *(E) or both MOs (F) shows ectopic neurogenesis between the medial and lateral proneural clusters of r2 and r4 (blue arrows in D-F, compare with white arrowheads in C) when Her8a and/or Her3 activities are blocked. Few "ectopic" *neurog1*-positive can sometimes be found between the vcc and mnr2; this is however highly variable between individuals and observed in both control and morphant embryos. A-F are dorsal views of flat-mounted embryos, anterior left. Abbreviations: black arrows indicate proneural clusters: IN: presumptive interneurons, MN: presumptive motoneurons, r2: rhombomere 2, r4: rhombomere 4, r2l: lateral neurons of rhombomere 2, r4l: lateral neurons of rhombomere 4, SN: presumptive sensory neurons, vcc: ventro-caudal cluster. **G. **RT-PCR analysis of *her8a *expression (left and middle panels) in embryos injected with *her8a*MO1 ("mo1") and *her8a*MO2 ("mo2") versus control embryos ("ctr"). Low levels of full length, normally spliced *her8a *transcripts are detectable in morphants (left and middle panels, arrows) while abnormally spliced transcripts including all or part of intron 1 become produced (stars). Expression of *βactin2*, used as RT-PCR control, is indentical in all samples (right panel). The scheme at the top indicates the genomic structure of *her8a*, the position of exons (E, purple) and introns (black bars), the binding sites of *her8a*MO1 and MO2 (red), the position of RT-PCR primers (blue arrows) and the length of amplified wild-type products (excluding introns) (blue bars).

Among *hes6*-like genes, we found that *her8a *was the only one with expression in the anterior neural plate at the 3-somite stage. At this stage, *her13 *was restricted to a portion of the presumptive lateral proneural clusters in the anterior spinal cord (Additional file 4, Figure S2). As shown previously, *hes6 *was found to be expressed in the tail bud and posterior paraxial mesoderm (Thisse and Thisse, 2004), and *her8.2 *was very weakly or not expressed (not shown). In contrast, following weak expression during gastrulation [26], *her8a *displayed strong expression in the neural plate from the tailbud stage onwards. At tailbud, *her8a *is expressed throughout the neural plate, with the exception of the eye field and the midline, with strongest intensity at presumptive midbrain and hindbrain levels (Figure 2A, bracket). At early segmentation stages, the domain of strong *her8a *expression overlaps with that of genes expressed in progenitor pools within the presumptive mid- and hindbrain, such as *her3 *(Figure 2B) and *her5 *(Figure 2C) (purple arrows). It also encompasses the proneural clusters located in the mid- and anterior hindbrain area (e.g. compare with *neurog1 *expression, Figure 3A). At 10 somites *her8a *is expressed in stripes in the hindbrain, with denser expression in rhombomeres (r) 1 and 3-5 (Figure 2D,E). Expression in the midbrain persists. From 24hpf onwards, *her8a *acquires a distinct expression profile, highlighting neurogenic domains throughout the central nervous system, with weaker staining at the midbrain-hindbrain boundary (mhb) (arrow) and at the zona limitans intrathalamica (Figure 2F) (asterisk). Cross sections of the brain at this stage reveal that *her8a *expression is confined to the progenitor, ventricular domain, largely complementary to the expression of the post-mitotic neuronal marker HuC/D (Figure 2G). From 48hpf through to adult, *her8a *remains expressed in proliferation/ventricular zones throughout the brain (Figure 2H,I, and data not shown).

### Gain of Her8a function inhibits neurogenesis

Mouse and Xenopus Hes6 proteins are known as positive regulators of differentiation [24,27]. In this context, the expression of *her8a *across both pro-neural and non-neurogenic domains was puzzling and prompted us to explore Her8a function.

In a first gain-of-function approach, embryos were injected with *her8a *capped mRNA encoding the full-length protein at the one cell stage. They were subsequently fixed and analyzed at 3 somites. We observed that *her8a *misexpression caused a complete loss of *neurog1 *expression throughout the embryo (Figure 3A,B) (77% of cases, n = 22). Co-labeling with *tp63 *(previously ΔNp63), which highlights the border of the epidermal ectoderm juxtaposed to the neural plate [28] (Figure 3A, white arrowheads), showed that the size or morphology of the neural plate were not affected (100% of cases, n = 22), suggesting that overexpressing Her8a specifically blocks neurogenesis without an effect on neural plate formation. This was confirmed by expression analyses for patterning markers such as *barhl2, her5 *and *her9*, which highlight distinct domains along the entire antero-posterior axis of the neural plate [11,29,30] (Additional file 5, Figure S3). Together, these results indicate that *her8a *is capable of inhibiting neurogenesis, at least at non-physiological concentrations, and this even across domains normally co-expressing *neurog1 *and *her8a *such as the proneural clusters of the mid- and anterior hindbrain (vcc, r2MN, r2l, R4MN, r4l).

### Her8a is required to maintain the proper neurogenesis pattern in rhombomeres 2 and 4 and acts as binding partner for Her3

To better appreciate the endogenous requirements for Her8a, we next turned to a loss-of-function approach. Embryos at the one-cell stage were injected with morpholinos (MO) directed against the donor splice site of *her8a *exon 1 (MO1), the acceptor splice site of *her8a *exon 2 (MO2), or the her8a ATG (MO3), and were analyzed at 3 somites. Reverse transcription PCR was used to reveal strong down-regulation of expression and abnormal splicing of *her8a *transcripts with both MO1 and MO2, whereas other genes, such as *βactin2*, remained unaffected (Figure 3G). These observations substantiate that *her8aMOs *lead to knock-down of *her8a *expression.

**Figure 4:**
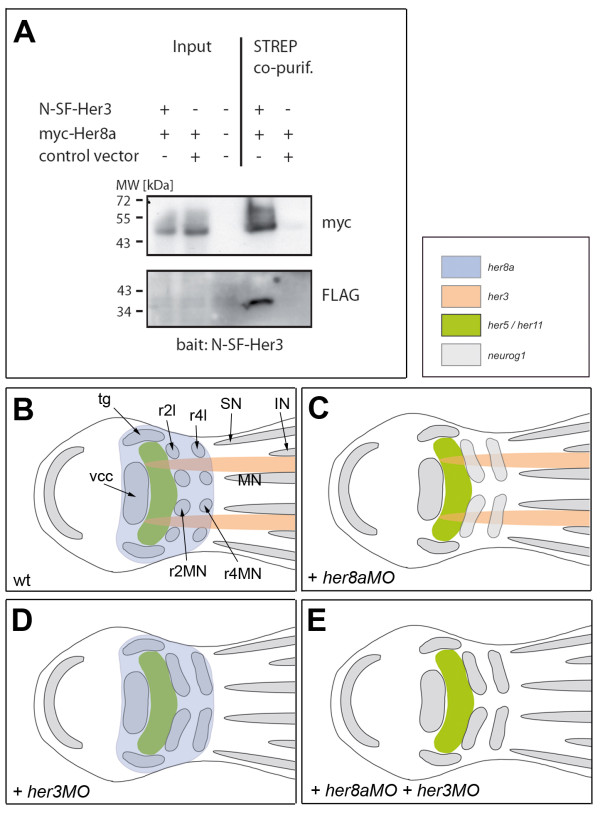
**The heterodimerisation potential of full-length Her8a and Her3 proteins may account for their identical loss-of-function phenotypes**. **A. **Co-affinity purification of full-length Her3 and Her8a proteins in HEK293T cells. When lysates from cells expressing both N-Strep/Flag-Her3 (N-SF-Her3) and Myc-tagged Her8a (myc-Her8a) proteins are eluted from a STREP-Tactin resin, myc-Her8a is observed to co-purify with N-SF-Her3 (lane 4). **B-E. **Schematized summary of the expression patterns (B) and loss-of-function phenotypes (C-E) of *her8a *and *her3*. Genes expression are color-coded and *her5/her11 *expression is indicated as a landmark (see also Figure 5H). Abbreviations: as in Figure 3; tg: trigeminal ganglion.

Blocking Her8a resulted in an ectopic expression of *neurog1 *within the normally non-neurogenic area separating motor- and lateral proneural clusters of r2 and 4 (Figure 3C, arrowheads, Figure 3D, arrows) (80% of cases, n = 60). Although the location of *neurog1*-positive cells can be slightly variable from embryo to embryo, this phenotype was robust and never observed in wild-type animals. The results obtained with the three MOs were strictly identical (Additional file 6, Figure S4 for a comparison of MO1 and MO2, and data not shown), confirming their specificity. We will compile these data below. This knockdown phenotype strikingly resembles the published effect of *her3 *knockdown [15], which we further confirmed (Figure 3E) (56% of cases, n = 50). Given that the r2/r4 area of ectopic neurogenesis is where the intense expression of *her8a *overlaps with that of *her3 *(Figure 1B, Figure 4B), these identical phenotypes suggest a direct or indirect functional interaction of Her3 and Her8a in controlling common progenitors. As a first obvious possibility, we tested whether *her3 *and *her8a *influence each other's expression. Ruling out this scenario, *her8aMO*-injected embryos displayed a normal expression of *her3*, and *her3MO *embryos a normal expression of *her8a *(not shown) (100% of cases, n = 37 and 24, respectively). A following hypothesis is that *her3 *and *her8a *act in a dose-dependent manner to compensate for each other outside r2 and r4, with r2 and r4 showing highest sensitivity to the amount of "Her3+Her8a" proteins. A similar situation was previously demonstrated for Her5+Her11 at the MHB [22]. In this case, we expect that the knockdown of both genes would produce a phenotype of a greater magnitude than the ectopic expression of proneural markers in r2 and r4. To test this possibility, we simultaneously blocked Her3 and Her8a by the co-injection of *her3MO *and *her8aMO *(at the same concentrations shown to produce the individual phenotypes). This produced no additional effect on *neurog1 *expression (Figure 3F) (80% of cases, n = 20). Likewise, the co-injection of *her3MO *and *her8aMO *in amounts just below their effective doses (0.75 mM for *her8a *and 0.375 mM for *her3*) induced no phenotype in r2 and r4 (not shown). Together, these observations indicate that these two factors alone are not compensating for each other to repress *neurog1 *in other areas of the embryo.

As a final alternative hypothesis, and based on the recovery of Her8a as binding to a Her bHLH domain in yeast cells, we tested whether Her8a and Her3 could act as necessary heterodimerisation partners. As no commercial antibodies are available for Her3 and Her8a, and attempts by our laboratory to have them manufactured failed, we chose a co-purification approach using tagged versions of the full-length zebrafish proteins recombinantly expressed in HEK293T cells. By purifying Strep/Flag-tagged Her3 via its Strep-tag II moiety, Myc-tagged Her8a was successfully co-purified (Figure 4A), demonstrating that both proteins interact with each other. This interaction may be relevant to the maintenance of the progenitor pools within r2 and r4, where both *her3 *and *her8a *are strongly expressed, and hereby account for the identical phenotypes of Her3 and Her8a loss of function (summarized in Figure 4B-E).

### In the absence of Her3, 5, 9 and 11 activity, endogenous Her8a alone is insufficient to preserve *neurog1*-free progenitor pools in the midbrain-hindbrain domain

**Figure 5:**
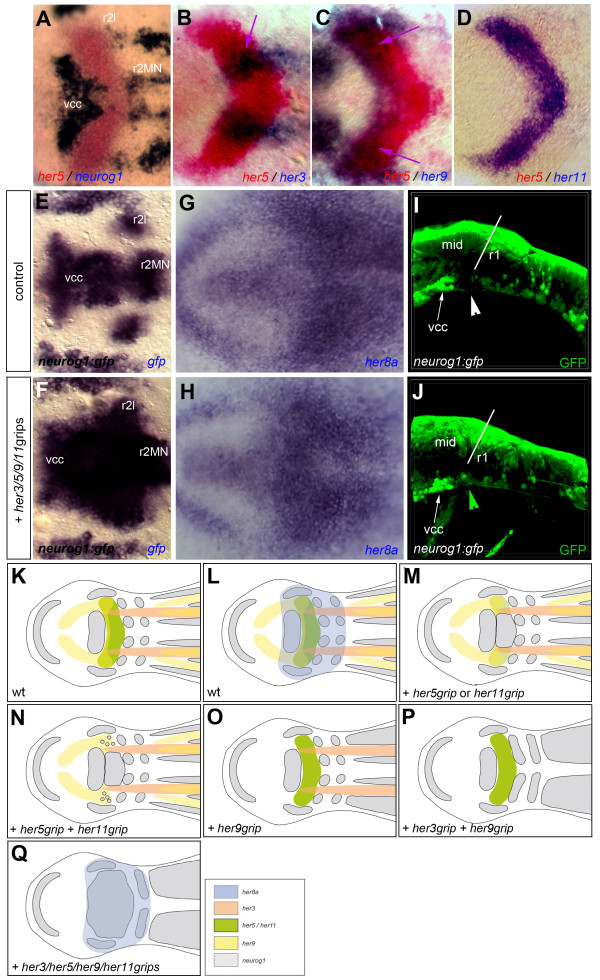
**The activities of Her3, Her5, Her9 and Her11 together account for the progenitor pool pattern of the midbrain-hindbrain area and do not influence *her8a *expression**. **A-D. **A comparison of the expression patterns of *her3, her5, her9, her11 *and *neurog1 *at 3 somites in the midbrain-hindbrain (MH) area using double in situ hybridization (color-coded), on dorsal views of flat-mounted embryos. Arrows point to the *her5/her11 *domains co-expressing *her3 *or *her9*. **E-J. **Compared neurogenesis in control embryos (E,G,I) and embryos co-injected with the four gripNA antisense oligonucleotides directed against *her3, 5, 9 *and *11 *transcripts (see Methods) (F,H,J). **E,F. **Expression of *neurog1 *(revealed by in situ hybridization against *gfp *in *-8.4neurog1:gfp *transgenic embryos) [62]: the majority of the MHB/r2 area is induced to express *neurog1*. **G,H. **The expression *her8a *is unaltered upon injection of the four gripNAs. **I,J. **Detection of GFP in *-8.4neurog1:gfp *embryos at 24 hpf (sagittal view, confocal projection of a 20 μm section of the neural tube). Ectopic neurons are formed ventrally across the midbrain-hindbrain boundary (position of the boundary indicated by the white bar), in a location normally devoid of GFP-positive cells (arrowheads). **K,L. **Summarized compared expression of *her3, 5, 9 *and *11 *(K), also together with *her8a *(L). **M-Q. **Summary of the combined loss-of-function results for MH-expressed *her *genes, from Geling et al. [13] (M), Ninkovic et al. [22] (N), Bae et al. [11] (O,P) and the present paper (Q). Abbreviations: mid: midbrain, MN: presumptive motoneurons, r: rhombomere, r2l: lateral neurons of rhombomere 2, vcc: ventro-caudal cluster.

The results above indicate that, although *her8a *is expressed across the entire MH domain, it is only strictly required to block neurogenesis in r2 and r4. This raises the question of which *her *genes combination encodes the endogenous pattern of neurogenesis inhibition in the MH domain, and whether this combination involves *her8a *expression. To address this issue, we used double in situ hybridization to re-analyze expression of the progenitor pools genes, comparing *her3, 5, 9 *and *11*. Our data confirmed the full overlap of *her5 *and *her11 *(Figure 5A,D) as well as the extension of *her3 *longitudinal stripes into the presumptive MHB domain [15] (Figure 5B, arrows), and revealed a previously unreported expression of *her9 *coinciding with the antero-lateral aspects of the *her5/11 *territory (Figure 5C, arrows) (summarized in Figure 5K,L). Previous loss-of-function experiments of combinations of these genes never achieved a full neurogenic phenotype: concomitantly blocking *her5 *and *her11 *induced *neurog1 *medially but only to a lesser extent in mediolateral and lateral MHB domains [22] (Figure 5M,N), and the co-inhibition of *her3 *and *her9 *largely recapitulated *her3 *loss-of-function in the MH area, with a restricted induction of *neurog1 *within r2 and r4 [11] (Figure 5O,P). We found that the down-regulation of all four factors together, through the coinjection of the relevant gripNA antisense oligonucleotides, was required to generate a large *neurog1*-positive domain across the presumptive MHB and r2 (Figure 5E,F) (74% of cases, n = 19) - although the most lateral aspects of the neural plate remained *neurog1*-free -. In these conditions however, *her8a *expression remained unperturbed (Figure 5G,H) (100% of cases, n = 39) (schematized in Figure 5Q). Together, these results demonstrate that the endogenous activity of Her8a, in the absence of other progenitor pools Her factors, is insufficient to inhibit *neurog1 *expression in the MH area. *her8a *expression also appears insensitive to the combined expression levels of Her3/5/9/11 and to the neurogenic status of this neural plate domain. When following the fate of ectopic *neurog1*-expressing progenitors in the absence of Her3/5/9 and 11 activities, we found however that only a subset were maintained until 24hpf. These were located ventrally across the midbrain-hindbrain boundary, immediately posterior to vcc-derived neurons (Figure 5I,J).

### Endogenous *her8a *expression in the early neural plate is independent of Notch signaling but requires the expression of SoxB factors

Although many E(spl) transcription factors are downstream effectors of Notch signalling, previous work has shown that zebrafish *her *genes expressed in progenitor pools, such as *her3, 5, 9 *and *11 *[11,13,31] exhibit a non-canonical regulation by Notch: they do not require Notch for their expression, and are insensitive to or transcriptionally inhibited upon ectopic Notch activation. This is in contrast to other family members such as *her4.1 *that are expressed in neurogenic zones and are activated by Notch signaling [32].

**Figure 6:**
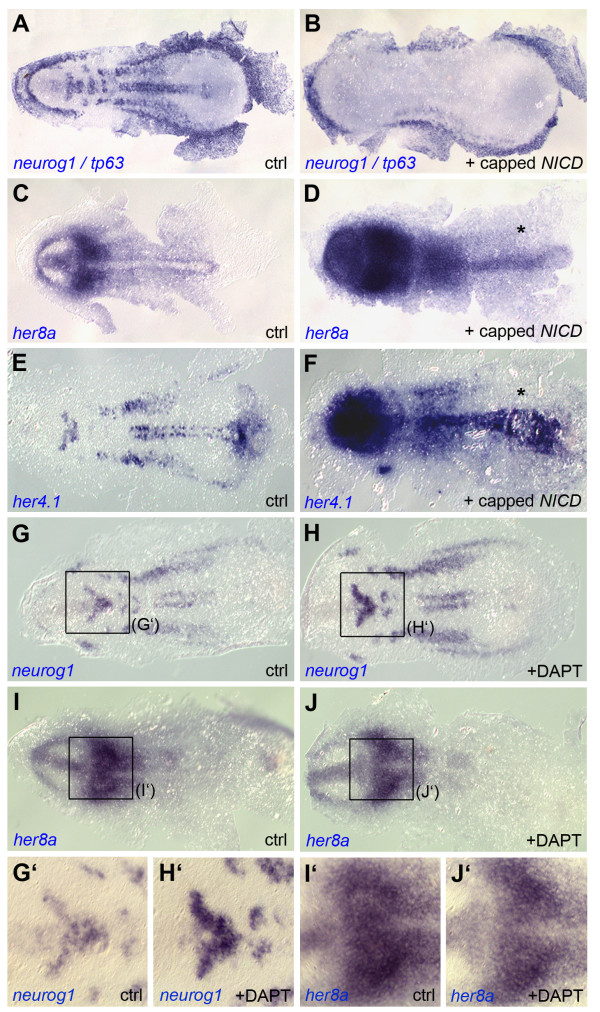
***her8a *expression is independent of endogenous Notch signaling in the early neural plate**. Dorsal views of flat-mounted embryos analyzed at the 3-somite stage for the expression of the genes indicated. **A-F. **Ectopic Notch activation (injection of capped *NICD *mRNA) abolished *neurog1 *expression (B) and activates *her8a *(D) and *her4.1 *(F) compared to non-injected embryos (ctrl). **G-J'. **Notch blockade (incubation in the gamma-secretase inhibitor DAPT) increases *neurog1 *expression within proneural clusters (H) but leaves *her8a *expression intact (J) at early neural plate stages compared to embryos treated with a vehicle only (ctrl). G'-J' are high magnification views of the areas boxed in G-J.

Unusually, *her8a *is expressed across both progenitor pools and proneural clusters in the early neural plate. To analyze the effect of ectopic Notch activation, we overexpressed the intracellular domain of zebrafish Notch1a (NICD) [10] through capped mRNA injection at the one-cell stage. We could replicate previously published results [10] showing that NICD misexpression completely downregulates neurogenesis throughout the early neural plate (Figure 6A,B) (88% of cases, n = 16). We found that overexpression of NICD causes ectopic or enhanced expression of *her8a *throughout the embryo (Figure 6C,D) (100% of cases, n = 17), although this induction was weaker at the caudal end of the neural plate (Figure 6D, asterisk). The latter observation was repeated when studying the *her4.1 *target gene (Figure 6E,F) (100% of cases, n = 13), suggesting a generally lower ability of this neural plate area to respond to NICD overexpression, rather than a *her8a*-specific feature in Notch response. Next, to determine whether *her8a *expression depends on endogenous Notch signaling, we incubated embryos between the 50% epiboly and 3-somite stages into the gamma-secretase inhibitor DAPT, which blocks Notch by preventing the cleavage of NICD and has a strong neurogenic effect [33,34]. As anticipated from previous studies [33], DAPT treatment increased the amount of *neurog1*-positive cells within each proneural cluster (Figure 6G-H>') (79% of cases, n = 19). However, it failed to reproducibly affect the expression of *her8a *(Figure 6C,I-J') (100% of cases, n = 15). Together, these results indicate that endogenous Notch signaling is not required for *her8a *expression in the early neural plate.

**Figure 7:**
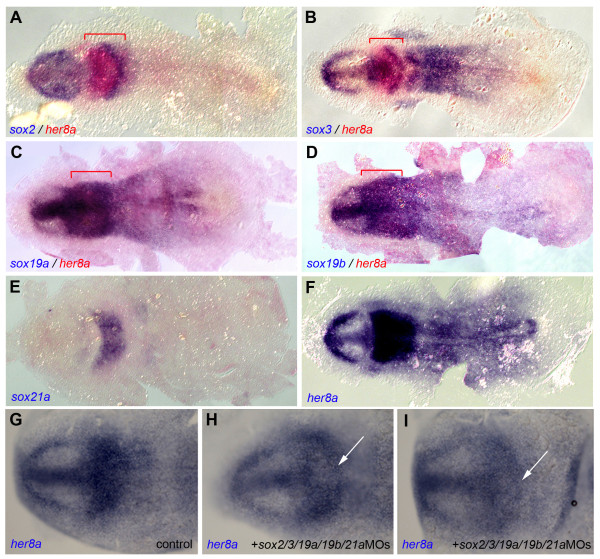
***her8a *expression in the early neural plate is partially dependent on the expression of SoxB factors**. Dorsal views of flat-mounted embryos analyzed at the 5-somite stage (A-F) and 3-somite stage (G-H) for the expression of the genes indicated (color-coded). **A-F. ***her8a *expression overlaps with that of the Sox family members *sox2, sox3, sox19a, sox19b *and *sox21a*, including the mid- and anterior hindbrain (red brackets). **G-I. **the simultaneous knockdown of *sox2/3/19a/19b *and *21a *causes a reduction of *her8a *expression in the MH area of the early neural plate (white arrows).

In order to gain further insight into the endogenous mechanisms controlling *her8a *regulation at these stages, we scanned the *her8a *promoter (100 bp downstream and 1000 bp upstream of the ATG start site) with the ModelInspector program (Genomatix) [35]. This revealed a potential Sox (Sry-related HMG box) binding site (SORY_OCT1_01) at position 873-897(+). ModelInspector uses Genomatix's in-house Promoter Module Library, which includes experimentally verified models for functional promoter subunits. In this case the promoter sequence was derived from a publication describing the activation of the mouse *Fgf4 *enhancer by Sox2 and Oct-3 [36]. This led us to investigate the possibility that a member of the *sox *gene family is controlling *her8a *expression. Mouse *Sox2 *is a member of Group B1, a subdivision of *Sox *genes involved in neural development [37]. Within this subgroup, we focused on the zebrafish genes *sox2 *[37], *sox3 *[37], *sox19a *and *sox19b *[38] (see [37] for phylogenetic description), excluding *sox1a *and *sox1b*, which are not expressed at the MHB at early embryonic stages [26,37]. In addition, we also investigated *sox21a *(previously *sox21 *or *sox30*), a member of the related subgroup B2 with specific MHB expression at early embryonic stages [39]. We found that expression of these different *sox *genes overlapped all or part of the *her8a*-positive domain at the 5-somite stage: *sox2 *and *sox3 *displayed strongest overlap with *her8a *expression in the anterior hindbrain (mostly r3) (Figure 7A,B), *sox21a *at the MHB (Figure 7E), while *sox19a *and *b *were intensely expressed throughout the MH (Figure 7C,D). In addition, *sox2, 3, 19a *and *19b *all displayed an expression identical to *her8a *in the presumptive telencephalon and ventral diencephalon, excluding the eye field (Figure 7A-D, compare with F). To analyze the role of these genes in controlling *her8a *expression, we used MOs targeting their ATG start site [40-43]. A single MO was used to inhibit *sox2 *and *3*, which share the sequence surrounding their ATG. When injected individually at the one-cell stage, none of these MOs produced a phenotype on *her8a *expression at the 3-somite stage (not shown) (n = 25). However, the combined knock-down of all five *sox *genes at once caused reduced *her8a *staining in the MH area (Figure 7G-I) (100% of cases, n = 18), indicating that these factors cooperate, possibly in a dose-dependent manner, to enhance *her8a *expression within the early neural plate. In a reverse step, we analyzed whether these genes were linked by a positive regulatory loop. We found however that blocking Her8a function upon *her8aMO *injection had no effect on *sox *genes expression at the 3-somite stage (not shown) (100% of cases, n = 10 for each *sox *gene tested).

## Discussion

### Her8a is a neurogenesis repressor in the early zebrafish neural plate

Two lines of evidence demonstrate that Her8a can act as a repressor of neurogenesis: firstly, the overexpression of full-length *her8a *causes a complete loss of *neurog1 *expression in the early embryo; secondly, we show that morpholino-mediated knockdown of *her8a *causes ectopic *neurog1 *expression in rhombomeres 2 and 4. These results are surprising, since the Hes6-like factors studied to date tend to exhibit neurogenesis-promoting activity. When ectopically expressed, Hes6 promotes neurogenesis in the Xenopus embryo [27], the differentiation of cortical neurons at the expense of astrocytes in the mouse [44,45], and the differentiation of retinal precursor cells into photoreceptors in mouse retinal explants [24]. These activities at least in part involve functionally antagonizing Hes1, since it was shown that Hes6 alone cannot bind the canonical E(spl) binding site (N box) [24,45]. Rather, Hes6 dimerises with Hes1 and modifies its DNA binding properties [24], its capacity for recruiting the co-repressor Groucho or its stability [44]. Interestingly at least some of these properties appear controlled by the loop domain of Hes6, which is five amino acids shorter than that of other E(spl) proteins (see Additional file 3, Figure S1). Indeed, the addition of five amino acid residues into the loop of Hes6 confers Hes1-like repressor activity on the N box, while conversely, the removal of five amino acid residues from the loop of Hes1 completely ceases repression activity and confers Hes6-like activity [24]. We observed that Her8a has an intermediate loop-length compared to Hes6 and Hes1 (Additional file 3, Figure S1). Although the functional significance of this feature remains a matter for investigation, it is possible that it confers specific mechanistic properties to Her8a that distinguish it from Hes6 and bring it closer to the mode of action of Hes1-like proteins. In addition, our phylogenetic and synteny analyses revealed the complex evolution of *Hes6*-like genes and, contrary to previous belief, that Her8a is not a direct ortholog of Hes6. Rather, Hes6 orthologs comprise zebrafish Her13 and Hes6, both of which have the same loop length as mouse Hes6. We show here that zebrafish *her13 *is specifically expressed in a pattern coincident with neurogenesis (Additional file 4, Figure S2) reminiscent of *Hes6 *expression in the developing nervous system of both mouse and Xenopus, highlighting committed progenitors or early neurons [24,27]. Thus we would predict that zebrafish Her13, rather than Her8a, shares functional properties with mammalian Hes6. The function of Hes6.2 subfamily proteins, to which Her8a belongs, has not been thoroughly tested, largely due to their absence in mammals. In the chicken neural tube however, Hes6.2 exerts a neurogenesis promoting activity [46], suggesting that Hes6.2 proteins may differ in their activities. We further propose that the splitting of *her8a *from locus 2 to a distinct genomic location (Figure 1C) permitted the acquisition of a unique expression profile for this gene in zebrafish.

### Combined Her activities generate the midbrain-hindbrain progenitor pool pattern through different modalities

Our loss-of-function studies demonstrate that Her8a activity is necessary to prevent neurogenesis within the mediolateral territory of r2 and 4, hence keeping the proneural clusters for moto- and lateral neurons spatially separated within the anterior hindbrain. Combined with the fact that endogenous *her8a *expression does not depend on Notch at this developmental stage (Figure 6), this function is typical of a "pre-patterning" activity, comparable to that exhibited by the other E(spl) factors Her3, 5, 9 and 11 that delimit the territories competent for *neurog1 *expression within the neural plate [11,13,15]. Despite this functional relevance, the phenotype of *her8a *morphants appears very restricted compared to the broad expression of *her8a*, which encompasses the entire mid- and anterior hindbrain (Figure 4B). Functional redundancy and dosage effects have been described for other members of the E(spl) family in the mouse neural tube [20] and the zebrafish early neural plate [11,22]. For example, Her5 and Her11 act in an equivalent and dose-dependent manner to block *neurog1 *expression in the medial and lateral aspects of the presumptive MHB [22], and Her3 and Her9 also cooperate to inhibit neurogenesis within the longitudinal stripe separating the presumptive moto- and interneuron clusters of the spinal cord [11]. We found that all *her *genes analyzed here (*her3, 5, 9, 11 *and *8a*) were at least partially co-expressed within the presumptive MH (Figure 5L), strongly suggesting that redundancy may account for the normal development of this area in *her8a *morphants. The situation in the hindbrain, however, appears different. The only two *her *genes highlighting progenitor pools in r2-4 are *her3 *and *her8a*. While morphant embryos for each of these genes have an identical phenotype, our results argue against a dose-dependent mechanism involving Her3 and Her8a. Indeed, we found that the co-injection of *her8aMO *and *her3MO *at active doses did not produce an additional phenotype (Figure 3F) and that, if both morpholinos were injected together in amounts just below their effective concentration, ectopic *neurog1 *expression was not observed. Given that the two factors do not regulate each other's expression, these experiments suggest that the presence of each factor individually, rather than their overall dose, is relevant to maintain neurogenesis inhibition within r2 and r4. Although Her8a was isolated as a binding partner for Her5 in yeast cells, co-purification shows that the full length Her8a and Her3 proteins heterodimerise (Figure 4A), and the overlapping expression of *her8a *and *her3 *makes it possible that this interaction occurs in vivo. Her proteins can dimerise with a variety of partners, as also supported by our yeast two-hybrid results (Additional file 1, Table S1), and Her-Her heterodimers display enhanced stability over homodimers [7,22]. A parsimonious interpretation of our results is therefore that the heterodimerisation of Her8a and Her3 is required for sufficient activity of these factors in r2 and r4. Alternatively, the individual activities of Her3 and Her8a may control complementary properties necessary to maintain the progenitor pool cell state.

In spite of the high level of *her8a *expression across the MHB progenitor pool, the results of the present paper also identify that the decisive inhibition of *neurog1 *expression in this location is played by other factors, namely Her3, 5, 9 and 11. Her5 and 11 were known for their dose-dependent redundant functions, accounting for neurogenesis inhibition across part of this domain [22]. Through knocking-down all four genes, we could achieve for the first time the transformation of most of the MHB into a neurogenic domain (Figure 5), while leaving *her8a *expression intact. Collectively, our findings show that the progenitor pool pattern of the midbrain and anterior hindbrain is established by the joined activities of five prepatterning E(spl) factors which act in different combination in the MHB and rhombomere domains. They also suggest that distinct mechanisms of action of these factors may be involved in these two domains.

Importantly however, we observed that the massive neurogenic phenotype induced upon blocking Her3/5/9/11 E(spl) activities is only partially followed by neuronal differentiation (Figure 5). In fact, ectopic neurons are restricted to the ventrolateral aspects of the midbrain-hindbrain boundary, like upon blocking Her5 function alone [21]. The corresponding progenitor population may be particularly prone to neuronal differentiation. For all other progenitors, our observations suggest the need for a further commitment event, independent of Her3/5/9/11 activities, to achieve neuronal differentiation following *neurog1 *induction. Blocking Notch signaling concomitantly to Her3/5/9/11 did not allow further neurogenesis progression (C. Stigloher, unpub.). Persistent *her8a *expression in this context may contribute to neurogenesis reversion, although it was not possible to evaluate this possibility as embryos blocked for the activities of all five E(spl) factors developed abnormally.

### *her8a *expression in the early neural plate is controlled by Sox transcription factors but not Notch signaling

Two types of *her *genes have been recently distinguished based on their Notch response profile: those acting as Notch mediators, depending on Notch signaling for their expression and overexpressed upon Notch activation, and "non-canonical" *her *genes endogenously independent of Notch and repressed when Notch is experimentally activated (reviewed in [12]). The former class comprises zebrafish *her4.1 *and *her15*, expressed in active neurogenic domains such as proneural clusters of the early neural plate [9-11]; the latter class is composed of *her3, 5, 9 *and *11*, expressed in progenitor pools [11,13,15,21,22]. Our results illustrate that *her8a *is unusual in its expression pattern, which overlaps both proneural clusters and progenitor pools. This property is shared with its ortholog *Hes6.2 *in chicken [46]. *her8a *also shows a distinctive response to Notch signaling among *hairy/E(spl) *genes within the early neural plate, since it endogenously does not depend on Notch signaling but responds positively to the experimental activation of Notch (Figure 6). In agreement with the latter observation, we could identify Su(H) binding sites in the upstream regulatory sequence of *her8a*. However, this potential appears not to be used within proneural clusters of the early neural plate, demonstrating that Her8a is not a mediator of lateral inhibition. This is also in agreement with its uniform rather than salt-and-pepper expression profile. We found nevertheless that overexpressing *her8a *abolishes *neurog1 *expression even in proneural clusters where the two genes are normally co-expressed. Although we cannot ascertain that high overexpression levels mimic endogenous Her8a activity, one hypothesis reconciling this different information is that Her8a function within proneural clusters may generally dampen *neurog1 *expression, contributing to the function of other Her factors in Notch-inhibited precursors, and ensuring a proper differentiation schedule in committed progenitors. Although not further analyzed in this paper, we noted also that *her8a *expression becomes dependent on Notch signaling at later developmental stages (K. Webb, unpublished).

Our analyses of *her8a *expression in morphant contexts for other Her factors did not highlight cross-regulations, although we found several consensus N and E boxes within the 600 bp upstream of the *her8a *start site. Previous work demonstrated the positive regulation of Xenopus *Hes6 *by proneural bHLH proteins, in particular Neurogenin [27]. Given the presence of E boxes on the *her8a *promoter, and the co-expression of *her8a *and *neurog1 *in proneural clusters, it will be interesting to test whether *her8a *expression is also positively controlled by proneural factors in these locations. The Ngn1/Hes6 cascade is positively reinforcing proneural activity in Xenopus [27], but our functional data would predict an opposite outcome for a Neurog1/Her8a regulation in zebrafish.

Finally, our results show that the levels of *her8a *expression are under control of a combination of SoxB1 and B2 factors (Sox2/3/19a/19b and Sox21a, respectively) that display intense and partially overlapping expression within the anterior neural plate (Figure 7). In a recent study, Okuda et al. [41] demonstrated that SoxB1 factors function redundantly to control several successive aspects of zebrafish nervous system development, including neural plate patterning and primary neurogenesis. Although single morphants do not harbor a visible phenotype, *her3 *expression fails to be induced in quadruple SoxB1 morphants, strongly suggesting that the four factors act redundantly to activate *her3 *transcription [41]. In a comparable manner, we found that individual SoxB1/B2 morphants display no phenotype, while *her8a *expression is reduced in the MH domain when all five SoxB1/B2 proteins are abolished. Although we have not tested all possible knock-down combinations, and in particular did not assess the individual relevance of Sox21a in the context of the quadruple knock-out for SoxB1 proteins, these results demonstrate that *her8a *expression levels are under control of the activity of at least partially redundant SoxB proteins. Expression of these factors is an integral part of the mechanisms patterning the early embryo [40,41], linking *her8a *expression with neural plate regionalization. The identification of a Sox2 binding site within the *her8a *enhancer, and the fact that all SoxB proteins recognize a similar binding motif in vitro, further suggests that part of this control may be direct. In support of this hypothesis, direct binding of SoxB1 factors onto the *her3 *enhancer has been demonstrated [41]. Like several other SoxB2 proteins, Sox21b was shown to act as a transcriptional inhibitor during dorsoventral patterning of the zebrafish gastrula [40] and generally promotes neurogenesis [47]. It can however act as an activator in other contexts [48], and its specific effect on MH neurogenesis and *her *genes needs to be directly evaluated. It was recently proposed that SoxB1 transcription factors and Notch cooperate through distinct mechanisms in their control of neurogenesis inhibition, including the inhibition of proneural protein activity and the transcriptional upregulation of *Hes/her *genes, respectively [49]. Our results and those of Okuda et al. [41] suggest yet another level of regulation, where SoxB proteins directly control the level of expression of some *her *genes. Whether this is limited to Notch-independent contexts, such as with the regulation of *her3 *and *her8a*, remains to be addressed.

## Conclusions

In this work, we identify the Hairy/E(spl) transcription factor Her8a as a local inhibitor of neurogenesis in the developing hindbrain. Specifically, we show that Her8a function, like Her3 [11], is required to generate the non-neurogenic progenitor pools normally separating the presumptive moto- and lateral neurons of r2 and 4. We demonstrate that Her8a is a binding partner for Her3 and we propose that this interaction may be functionally relevant in r2 and r4. We further show that Her8a alone is not sufficient to inhibit *neurog1 *expression in the presumptive MHB area; this event depends, in contrast, on the combined activities of four other E(spl) factors, Her3, 5, 9 and 11. Unlike canonical *E(spl) *genes, we demonstrate that *her8a *does not depend on Notch signaling for its expression at early neural plate stages, and we identify a combination of SoxB factors that together enhance *her8a *expression. Finally, using phylogenetic analyses, we show that Her8a belongs to a Hes6-like subfamily that was recently lost in the mammalian lineage. This observation provides a context for the strikingly divergent functions of Her8a from Hes6; Hes6, which was previously believed to be the mammalian ortholog of Her8a, displays proneural activity. Together, our results characterize the phylogeny, expression and functional cascades involving a new Her factor, and highlight the complex interplay of E(spl) proteins that generates the neurogenesis pattern of the zebrafish midbrain-hindbrain area.

## Methods

### Yeast Two-Hybrid Analysis

Yeast two-hybrid screening was performed by Hybrigenics, S.A., Paris, France (http://www.hybrigenics.com). The coding sequence for amino acids 20 to 201 of the *Danio rerio *Her5 protein (GenBank proteic accession number gi: 18858797)

(amino acid sequence DRINQSLETLRMLLLENTNNEKLKNPKVEKAEILESVVHFLRAEQASETDPFQITRVKRARTEES

DEDVESPCKRQSYHDGMRTCLLRVSNFITGKSHEFGQELEKACENIHKSHSRQVQLLSTPSLIEPQVHLYEDPSQQHLAHVQLSNS

CTPSGCSKLAQRTVPAMTSSPKQPVMLCDPV)

was PCR-amplified and cloned into pB29 as an N-terminal fusion to LexA (N-Her5-LexA-C). The construct was checked by sequencing the entire insert and used as a bait to screen a random-primed *Danio rerio *embryo (stages 18-20 hpf) cDNA library constructed into pP6. pB29 and pP6 derive from the original pBTM116 [50] and pGADGH [51] plasmids, respectively. 76 million clones (7.6 -fold the complexity of the library) were screened using a mating approach with Y187 (mata) and L40DGal4 (mata) yeast strains as previously described [52]. 280 His+ colonies were selected on a medium lacking tryptophan, leucine and histidine, and supplemented with 2 mM 3-aminotriazole to handle bait autoactivation. The prey fragments of the positive clones were amplified by PCR and sequenced at their 5' and 3' junctions. The resulting sequences were used to identify the corresponding interacting proteins in the GenBank database (NCBI) using a fully automated procedure. For each interaction, a Predicted Biological Score (PBS) was computed to assess interaction reliability. This score represents the probability of an interaction being nonspecific. PBS relies on two different levels of analysis; the algorithm and methods used in the calculation are described in detail in Formstecher et al. [53]. Briefly, at first a local score takes into account the redundancy and independency of prey fragments (i.e. the times the interaction was detected with different independent clones and whether it was detected with different or the same fragments), as well as the distribution of reading frames and stop codons in overlapping fragments. Thus, interactions detected with several and different fragments are ranked with a very high confidence score and interactions detected with a single independent fragment are ranked with a moderate confidence score. Secondly, a global score takes into account the interactions found in all the screens performed at Hybrigenics using the same library. This global score represents the probability of an interaction being nonspecific. For practical use, the scores were divided into four categories, from A (highest confidence) to D (lowest confidence). A fifth category (E) specifically flags interactions involving highly connected prey domains previously found several times in screens performed on libraries derived from the same organism. Finally, several of these highly connected domains have been confirmed as false-positives of the technique and are now tagged as F. The PBS scores have been shown to positively correlate with the biological significance of interactions [54,55].

### Gene ontology analysis

Gene ontology enrichment analysis was performed on the recovered yeast-2-hybrid candidates from the categories A, B and C using the AmiGO "Term Enrichment tool" [56] (available at http://amigo.geneontology.org/cgi-bin/amigo/term_enrichment), using the following settings: ZFIN database as a background set, the maximum p-value set at 0.05 and a minimum number of gene products of two.

### Sequence alignment, protein domain identification, phylogenetic and synteny analyses

Protein sequences were retrieved by using a tblastn search [57] against the non redundant database on NCBI or on Ensembl genomic data (current release of genomes, July 2010). For non-annotated sequences and to further support the expression of the predicted gene, a search for expressed sequence tags was also performed by tblastn on the EST database of the NCBI server. A list of all sequences used for the molecular phylogeny and their genomic locations is provided in Additional file 7, Table S3. Protein sequences were aligned using the ClustalX software. Only unambiguously aligned residues were retained to build the phylogenetic tree: namely the basic, HLH and Orange domains (see Additional file 3, Figure S1). Maximum likelihood phylogeny was constructed using PhyML (substitution model: JTT, number of substitution rate categories: 4; gamma distribution parameter estimated; proportion of invariable sites estimated) [58]. Branch support was estimated by approximate likelihood-ratio test (aLRT, SH-like) [59]. For the synteny analysis, the position and orientation of annotated genes surrounding *hes6 *family members was retrieved using the Ensembl genome browser.

### Zebrafish strains

Adult zebrafish were maintained using standard fish-keeping protocols and in accordance with institute guidelines for animal welfare (defined by the Regierung von Oberbayern and the Services Vétérinaires de l'Essonne). Wildtype (AB) embryos were obtained through natural matings and were staged according to Kimmel et al. [60].

### In situ hybridization

In situ hybridization on embryos was performed as previously described [22,61] using the following probes: *gfp *[62], *neurog1 *[63], *deltaNP63 *[64], *her4.1 *[10], *her5 *[29], *her9 *[65], *her3 *[11] and *barhl2 *[30]. For the *her8a *and *her13 *probes, 648bp and 785bp fragments (respectively) were cloned into pCRII-TOPO (Invitrogen) from cDNA from 24hpf AB embryos using the following primers: for *her8a *forward 5'CACTGCTTGGAAGCAAATGA34, reverse 5'GACTTGGCGTGTGATTGATG3' and for *her13 *forward 5'TTTCTGTCCAACCCCTTCTG3', reverse 5'GATCCAATCCGATGTTGCTT3' (PCR conditions available on request). The successful clones were verified by sequencing. RNA probes were synthesized following published protocols [66]. For immunohistochemisty the primary antibody was mouse Anti-*Hu *(diluted 1:1500) (*A21271; Molecular Probes) *revealed using Cy-2.

#### Whole-mount immunohistochemistry

GFP of the -*8.4neurog1:gfp *line [67] was detected using a chicken primary antibody (GFP-1020, Aves Labs, Inc) followed by a secondary antibody anti-chicken coupled to Alexa-488 (Molecular Probes, Invitrogen).

### RNA and morpholino injections

Capped RNAs were synthesized using Ambion mMessage mMachine Kit and embryos were injected at the one-cell stage. For *her8a *overexpression, full-length *her8a *was cloned by PCR (forward primer: 5'AATAATGACGGCCTCCAACA3'; reverse primer: 5'GGCTGCATTCATTCACCAG3') and cloned into pXT7. *her8a *capped mRNA was injected at a concentration of 62.5 ng/μl. NICD overexpression was achieved by injecting capped RNA for *nic*, which encodes the NICD fragment of zebrafish Notch1 [9,10]. Morpholinos were purchased from Gene-Tools (Philomath, USA) and gripNAs were purchased from Active Motif (Carlsbad, USA). Two *her8a *splice morpholinos were used, targeted against the donor and acceptor sites of *her8a *exons 1 and 2 (respectively *her8a*MO1 and MO2, see Figure 3G). Both *her8a*MO1 (ATGTGACATTACCTTTCGCTCCTCT) and MO2 (CGCAGCTAAAATGATAGAAAGCATG) were injected at 1mM. Their efficiency was evaluated by RT-PCR following standard protocols on pools of 25 embryos at the 3-somite stage, in comparison to *βactin2 *expression, using the following PCR primers (FL: primer designed to amplify the full-length her8 transcript; E2: primer designed to amplify the E1-E2 domain; FW: forward primer; REV: reverse primer): *her8a*-FL-FW: AATAATGACGGCCTCCAACA; *her8a*-FL-REV: GGCTGCATTCATTCACCAG; *her8a*-E2-REV: TCCTCTCTCTGCGTTTCTTCTC; *βactin2*-FWD: AAGGCCAACAGGGAAAAGAT; *βactin2*-REV: GTGGTACGACCAGAGGCATAC (expected size 109bp). A third *her8a *MO, targeted against the *her8a *ATG (MO3: CATTGCCCATGTTGGAGGCCGTCAT) was also used and injected at 1.5 mM. The *her3 *morpholino [15] was injected at 0.5mM. For the combined knockdown of *her3, her5, her9 *and *her11 *the following gripNAs were used, each at 0.4mM: *her3 *gripNA (AGCCATTGTCCTTAAATG) (overlapping the MO sequence published in [15]), *her5 *gripNA (GGTTCGCTCATTTTGTGT) [22], *her9 *gripNA (TGATTTTTACCTTTCTAT) (overlapping the MO sequence published in [11]) and *her11 *gripNA (AGTCGGTGTGCTCTTCAT) [22]. For the combined knock-down of *sox *genes, we used: *sox2/sox3 *morpholino (CTCGGTTTCCATACATGTTATACATT) [42,43] at 1mM (this MO was initially reported to target *sox2 *only, but we found that its target site is shared between *sox2 *and *sox3*), *sox19a *morpholino (TGCTGTACATGGCTGCCAACAGAAG) [41] at 1 mM, *sox19b *morpholino (TAGCCCTTTTCTCAAAACAAACCTG) at 0.25mM, *sox21a *morpholino (CATGGGCTTTGCCATTTCTTGATAC) (overlapping with the sox21aMO used in [40] at 1mM.

### DAPT treatment

DAPT treatment was carried out according to Geling et al. [33]. Embryos were placed in 100 μm DAPT (Alexis Biochemicals), 1% DMSO in embryo medium from 50% epiboly to 3 somites. Control embryos received a corresponding treatment with 1% DMSO. After treatment, the embryos were fixed in 4% PFA overnight at 4°C before being processed for in situ hybridisation.

### Co-affinity purification

The full length coding sequences of *her3 *and *her8a *were subcloned into the N-Strep/Flag (SF) TAP [68] and *myc pCS3+ *vectors, respectively. SF-TAP-tagged Her3 and Myc-tagged-Her8a were co-expressed in HEK293T cells. For this purpose 2 μg of *pCS3-myc-her8a *was co-transfected with either 6 μg *pcDNA3.0-SF-TAP-her3 *or control vector per 14 cm culture dish of HEK293T cells. After 48 h, cells were lysed in lysis buffer (TBS supplemented with Complete protease inhibitor (Roche), phosphatase inhibitor cocktails I and II (Sigma) and 0.5% NP-40 (Roche)). Beside the solubilization of cytoplasmic proteins, this condition allows the extraction of nuclear proteins which are not tightly bond to DNA. After incubation for 30 min at 4°C cell debris including nuclei were pelleted by centrifugation at 10,000 xg for 10 min, 4°C. The supernatants were filtered through 22-μm syringe filtration units (Millipore). Cleared lysates from two 14 cm culture dishes containing 5-6 mg total protein were incubated with 25 μl Strep-Tactin resin (IBA) for 2 h. After incubation the resin was washed 3 times with lysis buffer. The SF-TAP-tagged Her3 protein was eluted with 100 μl desthiobiotin elution buffer (IBA) and subjected to SDS PAGE. For detection, proteins were blotted onto PVDF membranes (GE Healthcare). The blots were incubated for 1 h with blocking solution (5% dry milk powder in TBST). The SF-TAP-tagged Her3 was detected by incubation of the blots with rabbit polyclonal anti FLAG antibodies (Sigma, 1:2000 in blocking reagent) overnight and secondary anti-rabbit antibodies (Jackson Immuno Research, 1:15,000 in blocking reagent) for 2 h. After each antibody incubation step, the blots were washed 4 times 5 min with TBST. For detection of Myc-Her8a, the blots were stripped using standard protocols and incubated with mouse anti-Myc (Cell signalling, 1:2000) overnight and secondary anti-mouse antibodies (Jackson Immuno Research, 1:15,000 in blocking reagent) for 2 h. Antibody-antigen complexes were visualized using the ECL+ chemiluminescence detection system (GE Healthcare) on Hyperfilms (GE Healthcare).

## Authors' contributions

KJW designed the two-hybrid assay, characterized the recovered proteins, identified *her8a *and carried out its initial functional characterization. She participated in the design of the study and helped to draft the manuscript. MC conducted the genomic and evolutionary analyses of Her8a, validated the efficiency of MO1 and MO2, conducted the functional characterization of Her8a using MO2 (including late fate analyses), and participated in the analyses of *her8a *regulation by Sox proteins. She drafted the relevant parts of the manuscript and provided critical input on the study as a whole. CJG developed the STREP/Flag assay in the laboratory of MU and conducted the co-purification experiments under his supervision and advice. He wrote the relevant sections of the manuscript. CS characterized the combined activities of *her3, 5, 9 *and *11 *and provided critical input during the study. BB and ST carried out the expression analyses in situ. LBC conceived the study, participated in its design and coordination, and drafted the manuscript together with KJW. All authors read and approved the manuscript.

## Supplementary Material

Additional file 1 **Table S1. Summary of yeast two-hybrid results**. The 75 unique protein-protein interactions recovered are listed, ordered according to their interaction scores (PBS: Protein Binding Score). The recovered *her *genes are underlined. Click here for file

Additional file 2 **Table S2. Gene Ontology enrichment analysis**. This analysis was conducted on the recovered yeast-2-hybrid candidates from the categories A, B and C. Click here for file

Additional file 3 **Figure S1. Alignment of mouse (Mm) Hes1, Hes5 and Hes6 with Hes6-like proteins from zebrafish (Dr) (Her8a, Her8.2, Her13 and Hes6) and other species (Fr: Fugu rubripes, Ol: Oryzias latipes, Gg: Gallus gallus, Hs: Homo sapiens, Xt: Xenopus tropicalis)**. Protein domains are color-coded: green: basic domain, purple: HLH domain, orange: orange domain. Note the signature of the Hes6-like subfamily: the loops of Her13 and Hes6 contain 5 less amino acids when compared with other members, and the loops of Her8a and Her8.2 have 3 less amino acids (domain overlayed with the black bar). In addition, the Orange domains of Hes6-like proteins are 65-86% identical to each other, while they show only 23-37% identity with the Orange domains of other Her/Hes proteins. Finally, similarities extending beyond the domains above also identify the Hes6.1 and Hes6.2 subfamilies within Hes6-like proteins (hatched red and blue boxes). Click here for file

Additional file 4 **Figure S2. *her13 *expression highlights early neurogenesis domains during zebrafish embryonic development**. Whole-mount in situ hybridization for *her13 *(blue staining) at the stages indicated (A-C and E are lateral views, D and F are dorsal views, all with anterior to the left). Note *her13 *expression in proneural clusters encompassing presumptive spinal interneurons (arrows) and sensory neurons (arrowheads), trigeminal ganglion neurons (asterisk), telencephlaic (t) and epiphyseal (e) neurons. Click here for file

Additional file 5 **Figure S3. Neural plate patterning is unaffected upon blocking Her8a function**. Whole-mount in situ hybridization for *barhl2 *(**A,B**), *her5 *(**C,D**) and *her9 *(**E,F**) in embryos injected with *her8a *capped mRNA (right column) compared to control embryos (left column). Dorsal views of whole-mount embryos are shown, anterior to the top. All three markers highlight defined neural plate territories (*barhl2*: transverse diencephalic domain; *her5*: prospective midbrain-hindbrain boundary; *her9*: prospective eye field, midbrain-hindbrain boundary and lateral rhombencephalic stripes, see Figure 5) and appear identically expressed in the wild-type and morphant neural plate. Abbreviations: d: diencephalon; e: eye field; mhb: midbrain-hindbrain boundary; sc: presumptive spinal cord. Click here for file

Additional file 6 **Figure S4. *her8a*MO1 and *her8a*MO2 have identical effects on *neurog1 *expression**. Whole-mount in situ hybridization for *neurog1 *expression in embryos injected with *her8a*MO1 (B) or *her8a*MO2 (C) compared to control embryos (whole-mount views of 3 somite-embryos, anterior to the top). *neurog1 *expression is ectopically induced between the clusters of motoneurons and lateral neurons in rhombomeres 2 and 4 (blue arrowheads in B,C), a location normally devoid of *neurog1 *transcripts (white arrowheads in A). The phenotype is highly reproducible and identical in both morphant groups. Click here for file

Additional file 7 **Table S3**. List of the sequences used for the molecular phylogeny (Figure 1) and their genomic locations. Click here for file
