# Supplementary material for: The Enhancer of split transcription factor Her8a is a novel dimerisation partner for Her3 that controls anterior hindbrain neurogenesis in zebrafish
Source: BMC Dev Biol. 2011 May 17;11:27. doi: 10.1186/1471-213X-11-27 (PMC3125270; doi:10.1186/1471-213X-11-27)
Supplement: Additional file 1 — Table S1. Summary of yeast two-hybrid results. The 75 unique protein-protein interactions recovered are listed, ordered according to their interaction scores (PBS: Protein Binding Score). The recovered her genes are underlined. [file 1471-213X-11-27-S1.DOC]

| **Supplementary table 1. Summary of yeast-2-hybrid results.** |
| --- |

| **Global PBS** | **Symbol** | **Name** | **GO terms - biological function; additional information** | **Accession no.** | **GID** |
| --- | --- | --- | --- | --- | --- |
| **A** | ***her8a*** | **hairy-related 8a** | **regulation of transcription** | **NM_199624.2** | **GID: 50878286** |
| A | *kpna4* | karyopherin alpha 4 (importin alpha 3) (kpna4) | protein import into nucleus | NM_201305.1 | GID: 41152017 |
| **A** | ***ppm1g*** | **protein phosphatase 1G (formerly 2C), magnesium-dependent, gamma isoform** | **protein amino acid dephosphorylation;**  **No functional data in zebrafish. Could be involved in the multiple cycles of phosphorylation and dephosphorylation required for splicing.** | **NM_201488.1** | **GID: 41393132** |
| **A** | ***psap*** | **prosaposin** | **sphingolipid metabolic process** | **NM_131883.1** | **GID: 18859264** |
| A | *apobl* | apolipoprotein B, like | lipid transport; response to chemical stimulus | NM_001030062.1 | GID: 71834285 |
| A | *pds5a* | PDS5, regulator of cohesion maintenance, homolog A | mitosis | XM_681303.1 | GID: 68354481 |
| B | *fam60a* | family with sequence similarity 60, member A |  | NM_198825.2 | GID: 52546699 |
| B | *her11* | hairy-related 11 | midbrain-hindbrain boundary development; negative regulation of neurogenesis; regulation of transcription | NM_001003886.1 | GID: 51468031 |
| **B** | ***hes6*** | **hairy and enhancer of split 6 (Drosophila)** | **regulation of transcription; somitogenesis** | **NM_194400.2** | **GID: 47271428** |
| B | hypothetical proteinXP_6950 | similar to LOC407638 protein | nucleotide and protein blast - no info; no conserved domains | XM_695066.1 | GID: 68437790 |
| B | *kpna2* | karyopherin alpha 2 (RAG cohort 1, importin alpha 1) | protein import into nucleus | NM_001002335.1 | GID: 50539737 |

| B | *mmp13* | matrix metalloproteinase 13 | metabolic process; proteolysis | NM_201503.1 | GID: 41393162 |
| --- | --- | --- | --- | --- | --- |
| **B** | ***her13* (previously zgc:110599)** | **hairy-related 13** | **regulation of transcription** | **NM_001017901.1** | **GID: 62955772** |
| B | *kpna5* | karyopherin alpha 5 (importin alpha 6) | protein import into nucleus | NM_001018153.1 | GID: 66392218 |
| **B** | ***ctnnbl1* (previously zgc:77673)** | **catenin, beta like 1** | **-; the function of this protein is yet to be determined. However, the C-terminal portion of the protein possesses apoptosis-inducing activity.** | **NM_200866.1** | **GID: 41053665** |
| C | similar to Lepre1 protein | similar to Lepre1 protein |  | XM_686014.1 | GID: 68440392 |
| **C** | **similar to Nucleoprotein TPR** | **similar to Nucleoprotein TPR** | **-; Contains TPR motifs, involved in protein-protein interactions. TPR motifs thought to be important for the functioning of chaperone, cell-cycle, transcription and protein transport complexes.** | **XM_694645.1** | **GID: 68390565** |
| D | *arntl1b* | aryl hydrocarbon receptor nuclear translocator-like 1b | signal transduction; photoperiodism; regulation of transcription | NM_178300.1 | GID: 30231255 |
| D | *cand1* | cullin-associated and neddylation-dissociated 1 |  | NM_213485.1 | GID: 47087304 |
| D | *dnmt7* | DNA (cytosine-5-)-methyltransferase 7 | DNA methylation | NM_001020476.1 | GID: 66472505 |
| D | *esrrd* | estrogen-related receptor delta | regulation of transcription | XM_685723.1 | GID: 68392616 |
| D | *her1* | hairy-related 1 | regulation of transcription; somitogenesis | NM_131078.1 | GID: 18858788 |
| D | *her12* | hairy-related 12 | Notch signalling pathway; brain development; regulation of transcription; somitogenesis | NM_205619.1 | GID: 45387662 |
| D | *her3* | hairy-related 3 | regulation of transcription | NM_131080.1 | GID: 18858792 |
| D | hypothetical proteinXP_6836 | PREDICTED: Danio rerio hypothetical protein LOC560265 | nucleotide and protein blast - no info; no conserved domains | XM_683661.1 | GID: 68390714 |
| D | hypothetical proteinXP_6873 | PREDICTED: Danio rerio hypothetical protein LOC563968 | nucleotide and protein blast - no info; no conserved domains | XM_687330.1 | GID: 68363075 |
| D | *pax3* | paired box gene 3 | regulation of transcription; xanthophore differentiation | NM_131277.1 | GID: 18859206 |
| D | *pax6a* | paired box gene 6a | regulation of transcription; hindbrain development; lens development in camera-type eye | NM_131304.1 | GID: 18859208 |
| D | *pax7* | paired box gene 7 | pigmentation during development; regulation of transcription | NM_131326.1 | GID: 24158479 |
| D | *ppp2r5e1* | protein phosphatase 2, regulatory subunit B (B56) | signal transduction | NM_194412.2 | GID: 41282171 |
| D | Zebra fish - hom. of hZNF9 | putative homolog of ZNF91-like Human ; [prey614210 - Zebra fish - hom. of hZNF9] |  | putative homolog of ZNF91-like Human ; [prey614210 - Zebra fish - hom. of hZNF9] |  |
| D | Zebra fish - hom. of hZNF9 | putative homolog of ZNF91-like Human ; [Zebra fish - hom. of hZNF9] |  | putative homolog of ZNF91-like Human ; [Zebra fish - hom. of hZNF9] |  |
| D | Zebra fish - hom. of hDGKD | putative homolog of Human DGKD ; [prey614354 - Zebra fish - hom. of hDGKD] |  | putative homolog of Human DGKD ; [prey614354 - Zebra fish - hom. of hDGKD] |  |
| D | Zebra fish - hom. of prey6 | no match; [prey614326 - Zebra fish - hom. of prey6] |  | no match; [prey614326 - Zebra fish - hom. of prey6] |  |
| D | Zebra fish - hom. of prey6 | no match; [Zebra fish - hom. of prey6] |  | no match; [Zebra fish - hom. of prey6] |  |
| D | *rars* | arginyl-tRNA synthetase | ATP binding | NM_200048.1 | GID: 41053406 |
| D | *sept2* | septin 2 | cell cycle | BC067625.1 | GID: 45709376 |
| D | similar to 26S proteasome n | similar to 26S proteasome non-ATPase regulatory subunit 11 (26S proteasome regulatory subunit S9) (26S proteasome regulatory subunit p44.5), transcript variant 2 |  | XM_703351.1 | GID: 68437954 |
| D | similar to Bullous pemphigo | PREDICTED: Danio rerio similar to Bullous pemphigoid antigen 1, isoforms 6/9/10 (Trabeculin-beta) (Bullous pemphigoid antigen) (BPA) (Hemidesmosomal plaque protein) |  | XM_693209.1 | GID: 68391675 |
|  |  | (Dystonia musculorum protein) (Dystonin) (LOC569802) |  |  |  |
| D | similar to DNAtopoisomerase | PREDICTED: Danio rerio similar to DNA topoisomeraseII_beta |  | XM_693293.1 | GID: 68391678 |
| D | similar to Zinc finger prot | PREDICTED: Danio rerio similar to Zinc finger protein 180 (HHZ168) |  | XM_687093.1 | GID: 68398626 |
| D | similar to Zinc finger protein | PREDICTED: Danio rerio similar to Zinc finger protein 35 (Zinc finger protein HF.10) |  | XM_688351.1 | GID: 68432892 |
| D | similar to ankyrin repeat d | similar to ankyrin repeat domain 15 |  | XM_691965.1 | GID: 68361831 |
| D | similar to carnitine palmit | carnitine palmitoyltransferase 1A |  | XM_684585.1 | GID: 68432320 |
| D | similar to diacylglycerol k | similar to diacylglycerol kinase, delta 130kDa isoform 1 |  | XM_688420.1 | GID: 68363095 |
| D | similar to high-mobility gr | PREDICTED: Danio rerio similar to high-mobility group 20A |  | XM_688329.1 | GID: 68405007 |
| D | similar to kinesin-associat | similar to kinesin-associated protein 3 |  | XM_684890.1 | GID: 68355885 |
| D | similar to microfilament an | Danio rerio similar to microfilament and actin filament cross-linker protein isoform a |  | XM_685588.1 | GID: 68392944 |
| D | similar to pentatricopeptid | PREDICTED: Danio rerio similar to pentatricopeptide repeat domain 1 |  | XM_693706.1 | GID: 68353971 |
| D | similar to sal-like4 | PREDICTED: Danio rerio similar to sal-like 4 |  | XM_696252.1 | GID: 68442392 |
| D | similar to slit homolog3 | PREDICTED: Danio rerio similar to slit homolog 3 |  | XM_680326.1 | GID: 68404770 |
| D | similar to zinc finger prot | PREDICTED: Danio rerio similar to zinc finger protein 569 |  | XM_690612.1 | GID: 68440488 |
| D | similar toCG31756-PA | PREDICTED: Danio rerio similar to CG31756-PA |  | XM_680049.1 | GID: 68404750 |
| D | similar to kaiso | similar to kaiso |  | XM_685882.1 | GID: 68374537 |
| D | smarcb1 | SWI/SNF-related matrix associated protein |  | NM_131448.1 | GID: 54262108 |
| D | Zebra fish - GenMatch | unknown; [prey614119 - Zebra fish - GenMatch] |  | unknown; [prey614119 - Zebra fish - GenMatch] | GID: 61673598 |
| D | Zebra fish - GenMatch | unknown; [prey614132 - Zebra fish - GenMatch] |  | unknown; [prey614132 - Zebra fish - GenMatch] | GID: 53748639 |
| D | Zebra fish - GenMatch | unknown; [prey614297 - Zebra fish - GenMatch] |  | unknown; [prey614297 - Zebra fish - GenMatch] | GID: 54888710 |
| D | Zebra fish - GenMatch | unknown; [prey614322 - Zebra fish - GenMatch] |  | unknown; [prey614322 - Zebra fish - GenMatch] | GID: 82617456 |
| D | Zebra fish - GenMatch | unknown; [prey614424 - Zebra fish - GenMatch] |  | unknown; [prey614424 - Zebra fish - GenMatch] | GID: 53748639 |
| D | Zebra fish - GenMatch | unknown; [prey614430 - Zebra fish - GenMatch] |  | unknown; [prey614430 - Zebra fish - GenMatch] | GID: 28412547 |
| D | zgc:100951 | zgc:100951 |  | NM_001003622.1 | GID: 57525637 |
| D | zgc:110443 | zgc:110443 | regulation of Rab GTPase activity | NM_001024394.1 | GID: 66773154 |
| D | zgc:73380 | zgc:73380 |  | NM_200811.1 | GID: 41387133 |
| D | zgc:76878 | zgc:76878 | intracellular protein transport; protein import into nucleus, docking | NM_200905.3 | GID: 47131213 |
| D | zgc:77244 | importin 7 (previously zgc:77244) |  | NM_207049.1 | GID: 46309466 |
| N/A | *eef1g* | eukaryotic translation elongation factor 1 gamma | translational elongation | NM_173263.1 | GID: 27545276 |
| N/A | *mta2* | metastasis associated 1 family, member 2 | regulation of transcription, DNA-dependent | NM_214695.1 | GID: 47550704 |
| N/A | similar to Importin alpha-1 | PREDICTED: Danio rerio similar to Importin alpha-1 subunit (Karyopherin alpha-1 subunit) (SRP1-beta) (RAG cohort protein 2) (Nucleoprotein interactor 1) |  | XM_696267.1 | GID: 68442406 |
| N/A | similar to glucocorticoid m | PREDICTED: Danio rerio similar to glucocorticoid modulatory element binding protein 2 |  | XM_692267.1 | GID: 68403010 |
| N/A | similar to leprecan1 | PREDICTED: Danio rerio similar to leprecan 1 |  | XM_682009.1 | GID: 68358901 |
| N/A | similar to transcription fa | PREDICTED: Danio rerio similar to transcription factor Her-8a |  | XM_688549.1 | GID: 68357359 |
| N/A | *snrp70* | U1 small nuclear ribonucleoprotein polypeptide A |  | NM_001003875.1 | GID: 51468001 |
| N/A | Zebra fish - GenMatch | unknown; [prey614342 - Zebra fish - GenMatch] |  | unknown; [prey614342 - Zebra fish - GenMatch] | GID: 61673602 |
| N/A | zgc:112226 | zgc:112226 | proteolysis | NM_001024409.1 | GID: 66912206 |
