# Supplementary material for: The Enhancer of split transcription factor Her8a is a novel dimerisation partner for Her3 that controls anterior hindbrain neurogenesis in zebrafish
Source: BMC Dev Biol. 2011 May 17;11:27. doi: 10.1186/1471-213X-11-27 (PMC3125270; doi:10.1186/1471-213X-11-27)
Supplement: Additional file 2 — Table S2. Gene Ontology enrichment analysis. This analysis was conducted on the recovered yeast-2-hybrid candidates from the categories A, B and C. [file 1471-213X-11-27-S2.DOC]

### Biological Process

| **GO Term** | **Aspect** | **P-value** | **Sample frequency** | **Background frequency** | **Genes** |
| --- | --- | --- | --- | --- | --- |
| GO:0006606 protein import into nucleus | P | 4.12e-06 | 3/12 (25.0%) | 8/15451 (0.1%) | *kpna5, kpna2 kpna4* |
| GO:0051170 nuclear import | P | 4.12e-06 | 3/12 (25.0%) | 8/15451 (0.1%) | *kpna5, kpna2 kpna4* |
| GO:0034504 protein localization to nucleus | P | 4.12e-06 | 3/12 (25.0%) | 8/15451 (0.1%) | *kpna5, kpna2 kpna4* |
| GO:0051169 nuclear transport | P | 2.10e-05 | 3/12 (25.0%) | 13/15451 (0.1%) | *kpna5, kpna2 kpna4* |
| GO:0006913 nucleocytoplasmic transport | P | 2.10e-05 | 3/12 (25.0%) | 13/15451 (0.1%) | *kpna5, kpna2 kpna4* |
| GO:0017038 protein import | P | 9.73e-05 | 3/12 (25.0%) | 21/15451 (0.1%) | *kpna5, kpna2 kpna4* |
| GO:0033365 protein localization to organelle | P | 3.61e-04 | 3/12 (25.0%) | 32/15451 (0.2%) | *kpna5, kpna2 kpna4* |
| GO:0006605 protein targeting | P | 5.19e-04 | 3/12 (25.0%) | 36/15451 (0.2%) | *kpna5, kpna2 kpna4* |
| GO:0006886 intracellular protein transport | P | 3.24e-02 | 3/12 (25.0%) | 142/15451 (0.9%) | *kpna5, kpna2 kpna4* |
| GO:0033036 macromolecule localization | P | 3.47e-02 | 4/12 (33.3%) | 390/15451 (2.5%) | *kpna5 kpna2 apobl kpna4* |
| GO:0034613 cellular protein localization | P | 4.20e-02 | 3/12 (25.0%) | 155/15451 (1.0%) | *kpna5, kpna2 kpna4* |
| GO:0070727 cellular macromolecule localization | P | 4.28e-02 | 3/12 (25.0%) | 156/15451 (1.0%) | *kpna5, kpna2 kpna4* |

### Cellular Component

| **GO Term** | **Aspect** | **P-value** | **Sample frequency** | **Background frequency** | **Genes** |
| --- | --- | --- | --- | --- | --- |
| GO:0005643 nuclear pore | C | 7.09e-05 | 3/12 (25.0%) | 19/15451 (0.1%) | *kpna5 kpna2 kpna4* |
| GO:0046930 pore complex | C | 7.09e-05 | 3/12 (25.0%) | 19/15451 (0.1%) | *kpna5 kpna2 kpna4* |
| GO:0005635 nuclear envelope | C | 2.96e-04 | 3/12 (25.0%) | 30/15451 (0.2%) | *kpna5 kpna2 kpna4* |
| GO:0044428 nuclear part | C | 1.69e-03 | 4/12 (33.3%) | 180/15451 (1.2%) | *hes6 kpna5 kpna2 kpna4* |
| GO:0005634 nucleus | C | 2.24e-03 | 8/12 (66.7%) | 1803/15451 (11.7%) | *her13 pds5a hes6 kpna5 kpna2 her8a her11 kpna4* |
| GO:0043227 membrane-bounded organelle | C | 2.53e-03 | 9/12 (75.0%) | 2552/15451 (16.5%) | *her13 pds5a hes6 kpna5 kpna2 her8a her11 psap kpna4* |
| GO:0043231 intracellular membrane-bounded organelle | C | 2.53e-03 | 9/12 (75.0%) | 2552/15451 (16.5%) | *her13 pds5a hes6 kpna5 kpna2 her8a her11 psap kpna4* |
| GO:0043229 intracellular organelle | C | 1.12e-02 | 9/12 (75.0%) | 3044/15451 (19.7%) | *her13 pds5a hes6 kpna5 kpna2 her8a her11 psap kpna4* |
| GO:0043226 organelle | C | 1.12e-02 | 9/12 (75.0%) | 3045/15451 (19.7%) | *her13 pds5a hes6 kpna5 kpna2 her8a her11 psap kpna4* |
| GO:0012505 endomembrane system | C | 3.66e-02 | 3/12 (25.0%) | 148/15451 (1.0%) | *kpna5 kpna2 kpna4* |

### Molecular Function

| **GO Term** | **Aspect** | **P-value** | **Sample frequency** | **Background frequency** | **Genes** |
| --- | --- | --- | --- | --- | --- |
| GO:0008565 protein transporter activity | F | 1.03e-03 | 3/12 (25.0%) | 45/15451 (0.3%) | *kpna5 kpna2 kpna4* |
| GO:0046982 protein heterodimerization activity | F | 2.37e-02 | 2/12 (16.7%) | 21/15451 (0.1%) | *hes6 her11* |

Webb et al., Suppl. Table 2
