# Supplementary figures and images for: The Enhancer of split transcription factor Her8a is a novel dimerisation partner for Her3 that controls anterior hindbrain neurogenesis in zebrafish
Source: BMC Dev Biol. 2011 May 17;11:27. doi: 10.1186/1471-213X-11-27 (PMC3125270; doi:10.1186/1471-213X-11-27)

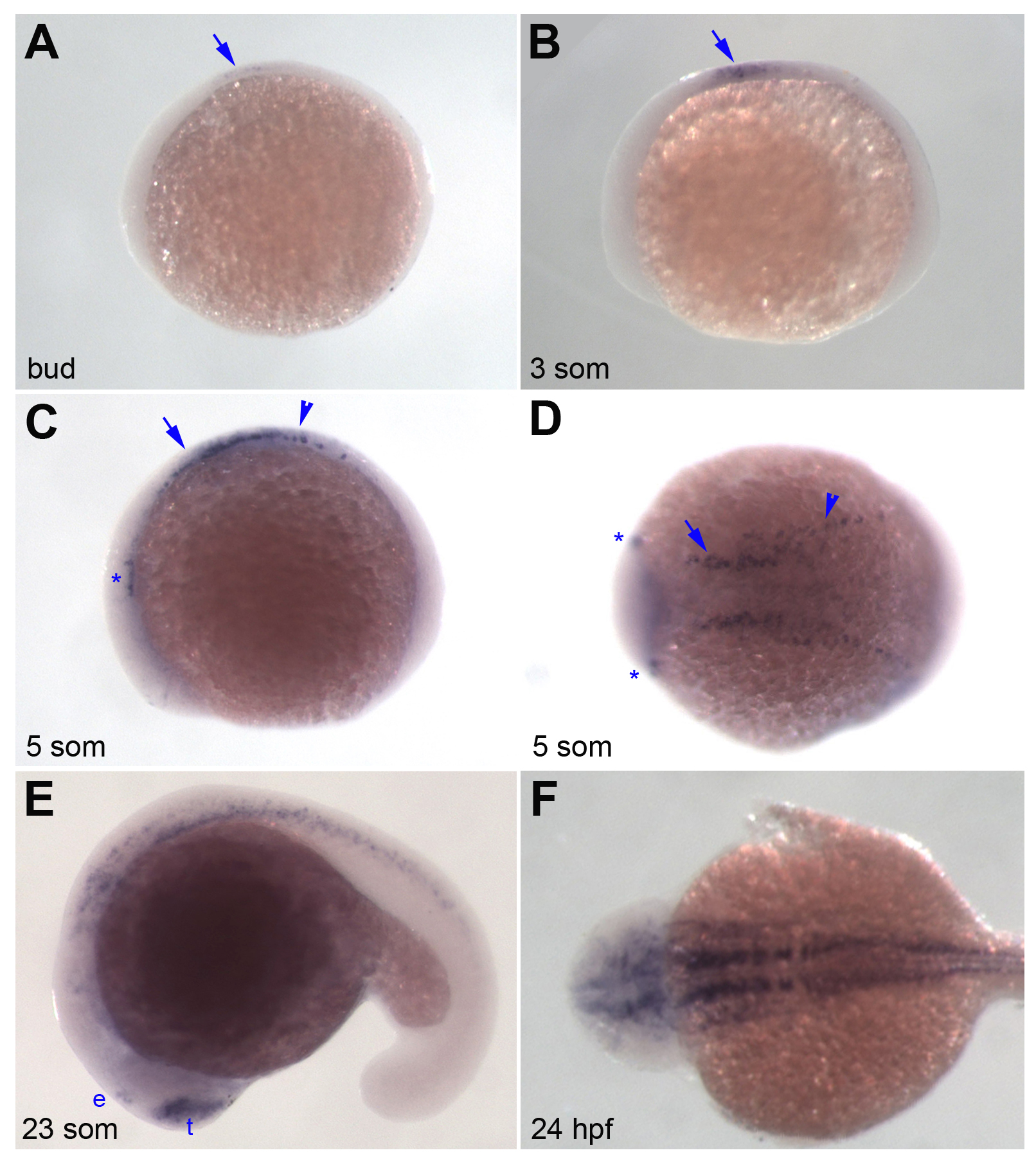

Supplement: Additional file 4 — Figure S2. her13 expression highlights early neurogenesis domains during zebrafish embryonic development. Whole-mount in situ hybridization for her13 (blue staining) at the stages indicated (A-C and E are lateral views, D and F are dorsal views, all with anterior to the left). Note her13 expression in proneural clusters encompassing presumptive spinal interneurons (arrows) and sensory neurons (arrowheads), trigeminal ganglion neurons (asterisk), telencephlaic (t) and epiphyseal (e) neurons. [file 1471-213X-11-27-S4.JPEG]

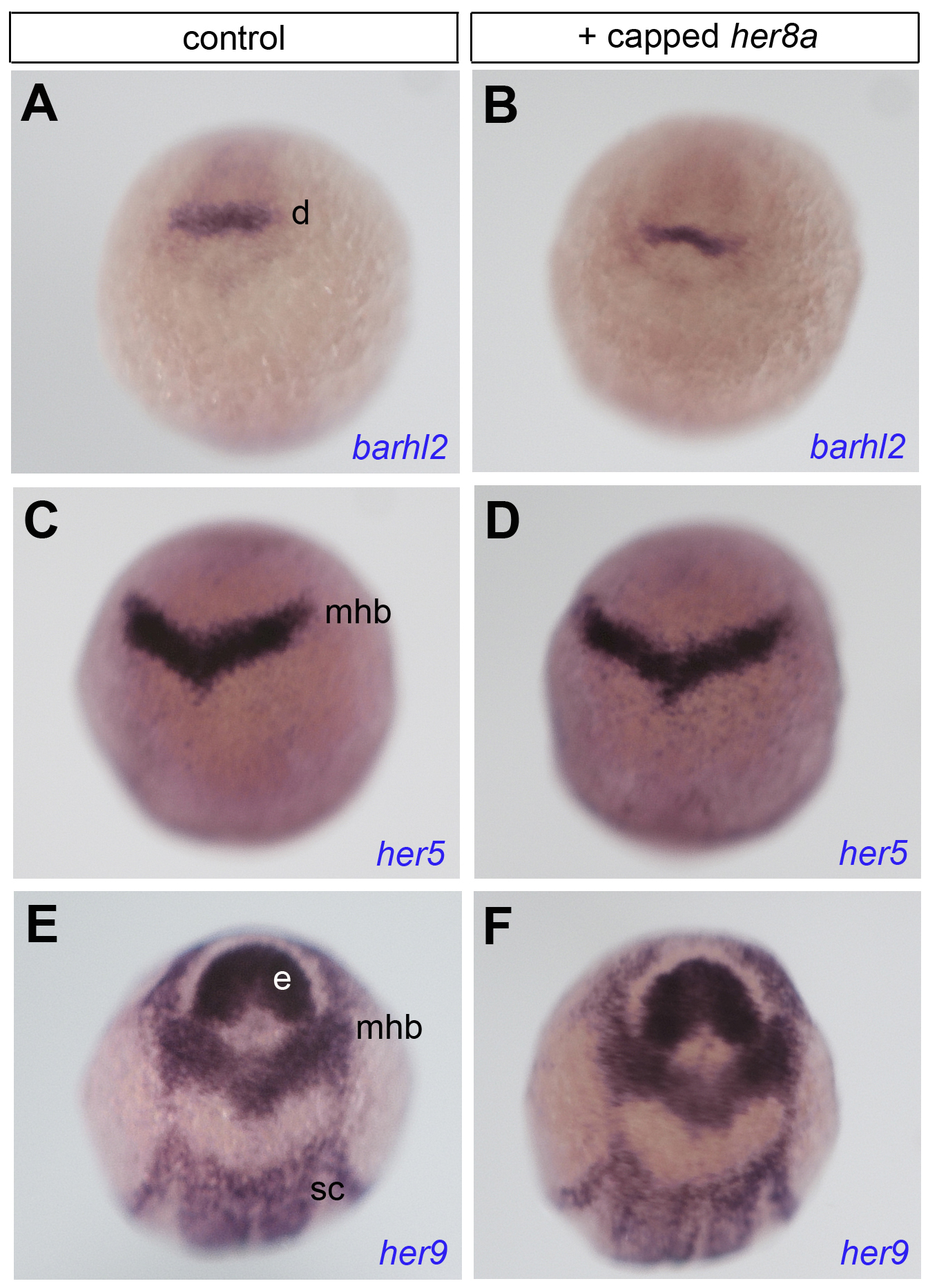

Supplement: Additional file 5 — Figure S3. Neural plate patterning is unaffected upon blocking Her8a function. Whole-mount in situ hybridization for barhl2 (A,B), her5 (C,D) and her9 (E,F) in embryos injected with her8a capped mRNA (right column) compared to control embryos (left column). Dorsal views of whole-mount embryos are shown, anterior to the top. All three markers highlight defined neural plate territories (barhl2: transverse diencephalic domain; her5: prospective midbrain-hindbrain boundary; her9: prospective eye field, midbrain-hindbrain boundary and lateral rhombencephalic stripes, see Figure 5) and appear identically expressed in the wild-type and morphant neural plate. Abbreviations: d: diencephalon; e: eye field; mhb: midbrain-hindbrain boundary; sc: presumptive spinal cord. [file 1471-213X-11-27-S5.JPEG]

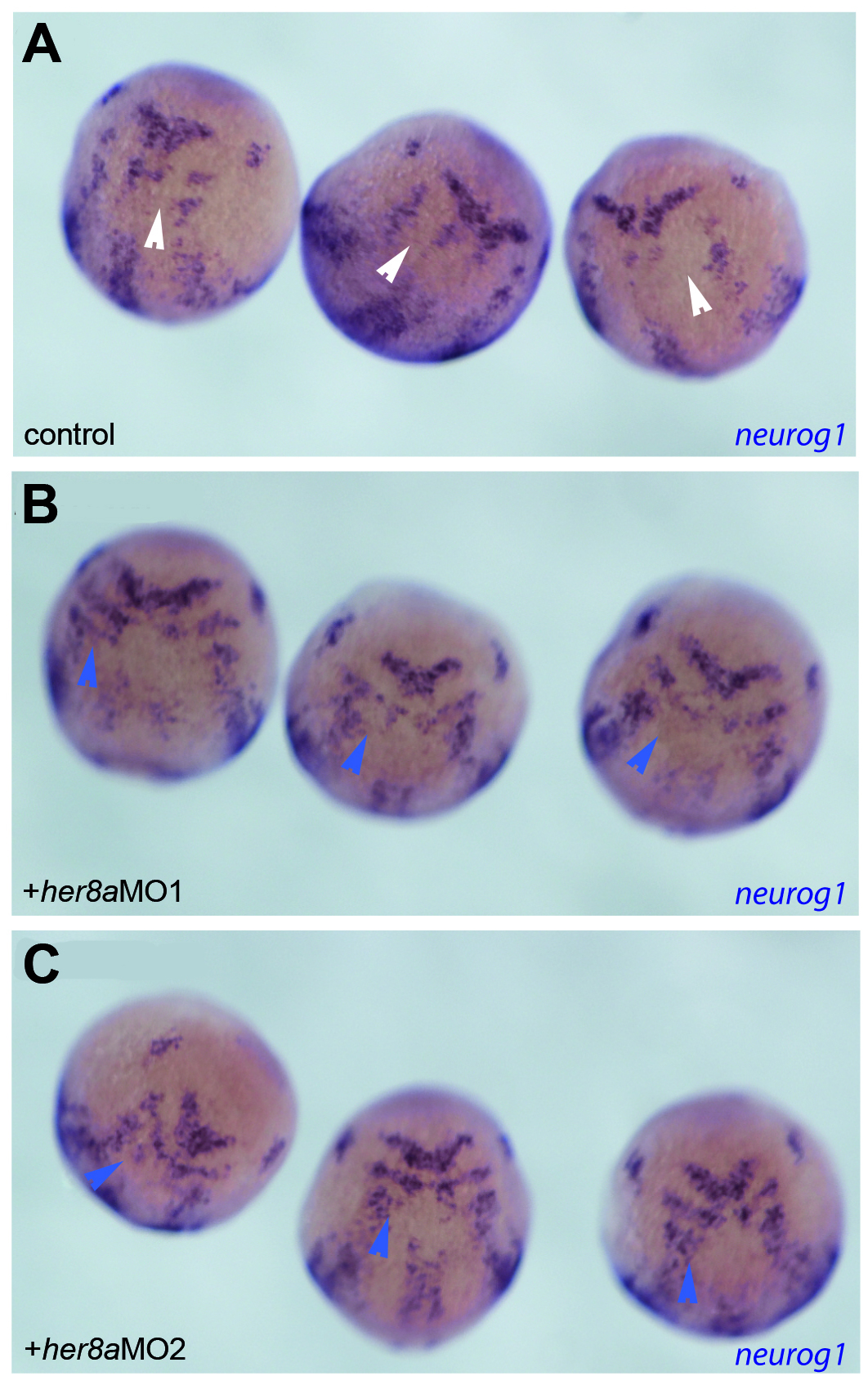

Supplement: Additional file 6 — Figure S4. her8aMO1 and her8aMO2 have identical effects on neurog1 expression. Whole-mount in situ hybridization for neurog1 expression in embryos injected with her8aMO1 (B) or her8aMO2 (C) compared to control embryos (whole-mount views of 3 somite-embryos, anterior to the top). neurog1 expression is ectopically induced between the clusters of motoneurons and lateral neurons in rhombomeres 2 and 4 (blue arrowheads in B,C), a location normally devoid of neurog1 transcripts (white arrowheads in A). The phenotype is highly reproducible and identical in both morphant groups. [file 1471-213X-11-27-S6.JPEG]

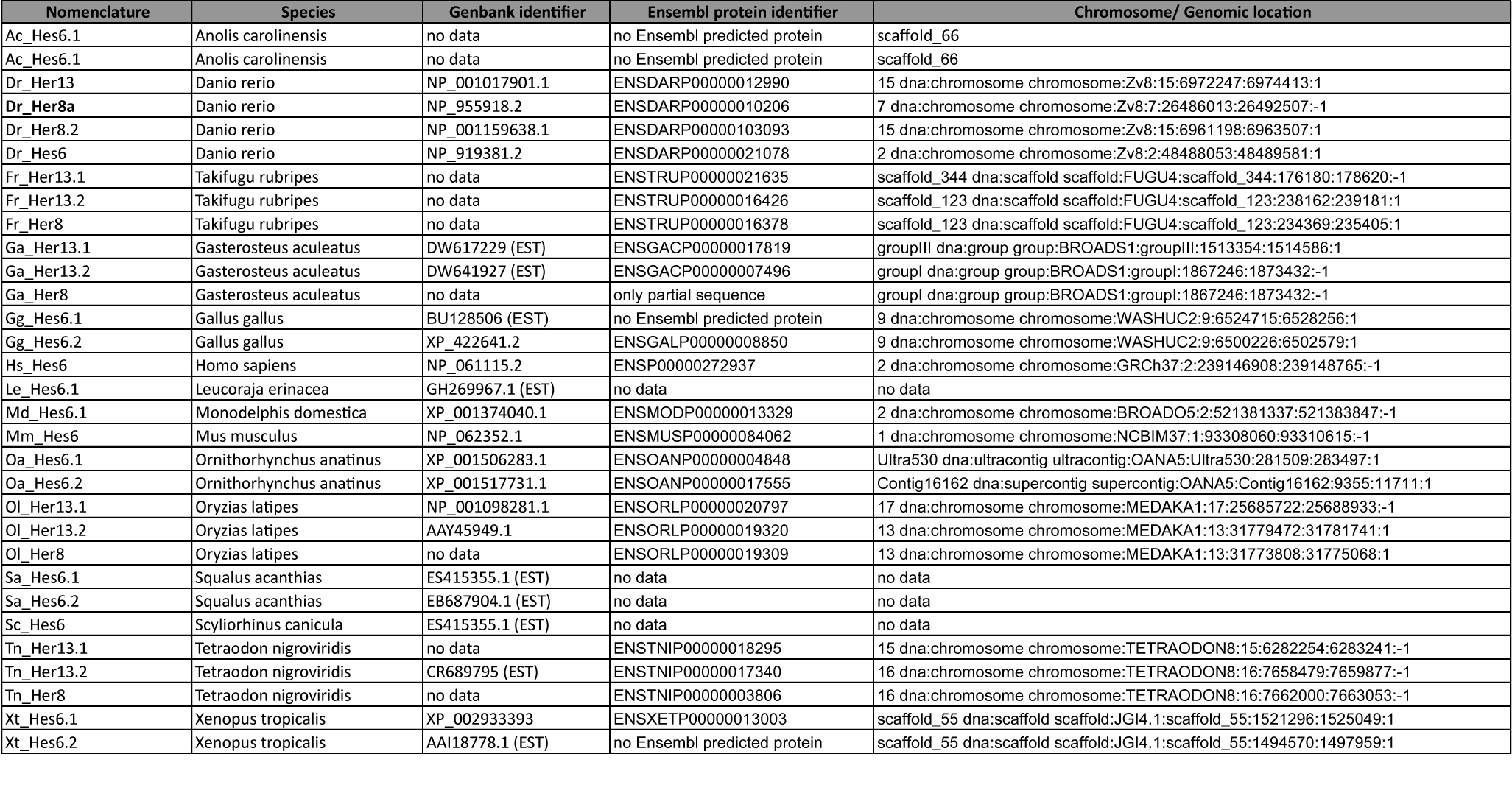

Supplement: Additional file 7 — Table S3. List of the sequences used for the molecular phylogeny (Figure 1) and their genomic locations. [file 1471-213X-11-27-S7.JPEG]
