# Supplementary material for: The Enhancer of split transcription factor Her8a is a novel dimerisation partner for Her3 that controls anterior hindbrain neurogenesis in zebrafish
Source: BMC Dev Biol. 2011 May 17;11:27. doi: 10.1186/1471-213X-11-27 (PMC3125270; doi:10.1186/1471-213X-11-27)
Supplement: Additional file 3 — Figure S1. Alignment of mouse (Mm) Hes1, Hes5 and Hes6 with Hes6-like proteins from zebrafish (Dr) (Her8a, Her8.2, Her13 and Hes6) and other species (Fr: Fugu rubripes, Ol: Oryzias latipes, Gg: Gallus gallus, Hs: Homo sapiens, Xt: Xenopus tropicalis). Protein domains are color-coded: green: basic domain, purple: HLH domain, orange: orange domain. Note the signature of the Hes6-like subfamily: the loops of Her13 and Hes6 contain 5 less amino acids when compared with other members, and the loops of Her8a and Her8.2 have 3 less amino acids (domain overlayed with the black bar). In addition, the Orange domains of Hes6-like proteins are 65-86% identical to each other, while they show only 23-37% identity with the Orange domains of other Her/Hes proteins. Finally, similarities extending beyond the domains above also identify the Hes6.1 and Hes6.2 subfamilies within Hes6-like proteins (hatched red and blue boxes). [file 1471-213X-11-27-S3.PDF]

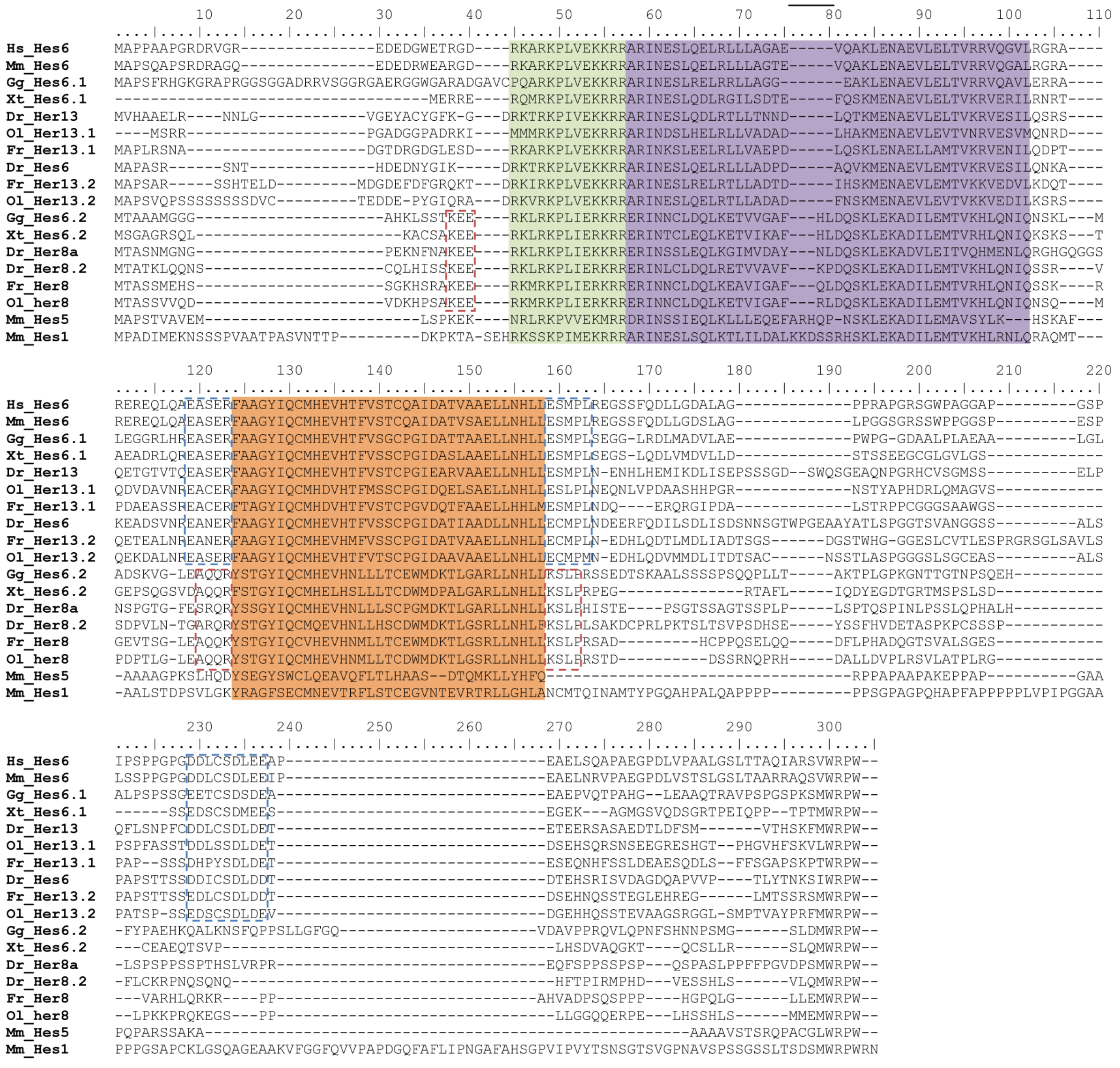
Hs_Hes6
Mm_Hes6
Gg_Hes6.1
Xt_Hes6.1
Dr_Her13
Ol_Her13.1
Fr_Her13.1
Dr_Hes6
Fr_Her13.2
Ol_Her13.2
Gg_Hes6.2
Xt_Hes6.2
Dr_Her8a
Dr_Her8.2
Fr_Her8
Ol_her8
Mm_Hes5
Mm_Hes1
